# Application of Capillary Electrophoresis for Determination of Inorganic Analytes in Waters

**DOI:** 10.3390/molecules26226972

**Published:** 2021-11-18

**Authors:** Ewa Poboży, Marek Trojanowicz

**Affiliations:** 1 Faculty of Chemistry, University of Warsaw, Pasteura 1, 02-093 Warsaw, Poland; trojan@chem.uw.edu.pl; 2 Laboratory of Nuclear Analytical Techniques, Institute of Nuclear Chemistry and Technology, Dorodna 16, 03-195 Warsaw, Poland

**Keywords:** capillary electrophoresis, inorganic ions, water analysis

## Abstract

Aside from HPLC and GC, capillary electrophoresis (CE) is one of the most important techniques for high-performance separations in modern analytical chemistry. Its main advantages are the possibility of using different detection techniques, the possibility of in-capillary sample processing for preconcentration or derivatization, and ease of instrumental miniaturization down to the microfluidic scale. Those features are utilized in the separation of macromolecules in biochemistry and in genetic investigations, but they can be also used in determinations of inorganic ions in water analysis. This review, based on about 100 original research works, presents applications of CE methods in water analysis reported in recent decade, mostly regarding conductivity detection or indirect UV detection. The developed applications include analysis of high salinity sea waters, as well as analysis of other surface waters and drinking waters.

## 1. Introduction

The current common requirement for the quality control of water for municipal and industrial needs, and also for environmental protection, has resulted practically in a permanent search for the development of improved analytical techniques and instrumentation for the analytical techniques used for the determination of inorganic macro- and micro-components, as well as organic compounds usually occurring at trace levels. With very wide needs for such analyses, the development of analytical techniques providing the possibility of simultaneous multicomponent determination is especially valuable. Atomic emission spectroscopy techniques were employed quite early for the purpose of determinations of numerous light elements, but the most significant breakthrough for both inorganic ions and organic analytes was the introduction of liquid chromatography techniques to the arsenal of water analysis techniques. The invention of high-performance ion-chromatography (IC) in the 1980s essentially improved determinations of inorganic ions in waters. Currently those IC methods are commonly accepted and routinely used in water analyses for various needs.

Considering both speed of analysis and the possibility of multicomponent determinations in IC determinations of anions and cations in waters, the only technique competitive with IC seems to be the capillary electrophoresis (CE). CE can be generally considered a technique complementary to IC in the determination of small inorganic ions due to its favorable features, such as short analysis times, relatively low running and instrumentation costs, small sample volume and reagent consumption, excellent separation efficiency, suitability for miniaturization, and portability. CE is also able to tolerate injection of samples with heavy loaded matrices as the capillary can be easily cleaned between injections. On the other hand, IC equipment and columns for ion chromatography are usually more expensive, and may have rather short lifetime.

Since its invention and employment in the first works of Virtanen in 1974 [1], and Jacobson and Lucas in 1981 [2], CE is considered an attractive and complementary separation technique to high-performance liquid chromatography (HPLC). This is very well documented by a very vast literature: nearly 1800 original research papers and review articles are now published annually. See, for instance, a review on current state of development and recent trends in analytical capillary electrophoresis [3]. One can notice, however, that although the main mechanism of CE deals with the separation of ions, in fact only a very small part of research efforts are focused on optimizing the separation of inorganic ionic species, while the majority of applications deal with determinations of organic analytes. This is a consequence of the limitations of commercially available CE detectors other than UV-VIS spectrophotometers. Numerous other detection techniques are laboratory adapted for research purposes, but not for routine use. So, in cases of routine applications for determination of ions which do not absorb UV-VIS radiation, indirect UV-VIS detection or laser-induced fluorescence (LIF) are employed, requiring appropriate derivatization of the analytes used.

Numerous studies have compared the detection limits of CE and IC in determinations of inorganic ions. For instance, in determinations of anions in drinking water, LODs for a single-column ion chromatography with conductivity detection were found in a ppm range, which for CE with indirect UV detection was about 0.3 to 0.5 ppm [4]. Detection of LODs for inorganic ions in a suppressed IC with conventional conductivity are similar to those of CE with indirect UV detection [5]. It has to be admitted, however, that additional in-capillary preconcentration, e.g., by so called stacking procedures, may significantly improve the detection limits of CE methods [6]. Without stacking preconcentration, however, the LODs for anions in IC systems with UV detection are lower than those for CE systems with indirect UV detection. 

The application of C^4^D contactless conductivity detection in CE significantly improves the limits of the detection of inorganic cations and anions, which are only 2.7 times higher than non-suppressed single column ICs with conductivity detection, and 10 times higher than those obtained for suppressed IC with conductivity detection, respectively [7]. It was shown, for instance, that for trace determination of Fe(II) and dissolved inorganic phosphorus in sediment porewater, the LOD values for IC with conductivity detection were insufficient, while the values obtained for CE with C^4^D detection were satisfactory [8].

Several other advantages of CE in comparison to IC should be also mentioned here, such as a better separation efficiency and resolution, the possibility of simultaneous determination of cations and anions in one run and in one capillary, easy miniaturization of instrumentation, and the possibility of the application of different efficient methods for the in-capillary preconcentration of analytes. The appropriate selection of the background electrolyte (BGE) composition may reduce the interfering effects of matrix components, which can affect the selectivity of the determination.

The subject of this review is to present recent achievements in the uses of CE with various detection methods and with different steps of sample processing in the determination of inorganic anions in waters, based on papers published in recent decades. The CE determinations of inorganic ions were already a subject of numerous publications in the past, including numerous reviews, see e.g., [9,10,11], and one on capillary isotachophoresis [12]. Several reviews on the application of CE techniques for the determination of inorganic ions in water analysis [13,14,15] and the use of portable CE systems for applications including water analysis [16] have also been published.

## 2. Basic Modes of Analytical Capillary Electrophoresis Measurements

Electrophoresis has for more than 200 years been known as the phenomenon of the transport of electrically charged molecules in a liquid or gel phase induced by an electric field. The electrophoretic mobility of ions in a background electrolyte (BGE) filling the capillary is directly proportional to the charge of the ions and inversely proportional to their size. This behavior of ions has been exploited for analytical purposes for many decades. Electrophoretic separations in planar layers of appropriate gels are very commonly used in biochemistry, and also for preparative purposes. In capillary electrophoresis as analytical technique, separation is usually carried out in capillaries of internal diameters of 25 to 75 μm, most often fabricated of fused silica, where the electric field is generated by the application of a high voltage (usually kilovolts) to electrodes which are immersed in solutions, into which two ends of the separation capillary are also immersed. The detection of ions are moving along the capillary is carried out by the detector located at the end of capillary. In CE, the electrophoretic migration of ions is also often associated with the electroosmotic flow (EOF) of the bulk solution within the capillary as the result of the accumulation of electric charge in the solution layer, in the inner capillary surface. The existence, magnitude and direction of EOF under particular conditions, depends on the composition of BGE. For analytical purposes, capillary electrophoretic separations and determinations can be carried out using different methods to provides the efficient separation and determination of electrically charged ions and neutral molecules.

Capillary zone electrophoresis (CZE) is the most commonly employed CE methodology for analytical purposes, where a portion of solution to be analyzed is introduced into the capillary, which is filled with the BGE. The EOF is generated in the whole capillary diameter with the same rate, providing a flat shape of the front of the solution (in contrary to parabolic one in laminar flow). This behavior is crucial to obtain efficient, high-performance separations with chromatographic efficiency close to that of gas chromatography separations. This methodology is the most commonly used for CE determinations of inorganic ions, which is the subject of this review.

Micellar electrokinetic capillary chromatography (MEKC) is based on the use of surface-active additives in BGE, in concentrations sufficient for the formation of micelles. This results in the formation of a so-called pseudostationary phase in the BGE, which creates the possibility of a partition of the analytes between the phase of the micelles and that of the BGE solution. This partition can take place for both charged and non-charged analytes, offering the conditions for capillary chromatographic separation.

Gel capillary electrophoreses (GCE) is carried out in capillaries filled with gels of appropriate pore sizes, and it is mainly employed for the separation of macromolecules. A porous gel phase acts as a molecular sieve, enabling separations according to the sizes of the separated macromolecules.

Capillary isotachophoresis (CITP) is the electrophoretic separation technique carried out with the use of a non-uniform electrolyte filling the separation capillary. The sample segment introduced into the capillary is placed between two zones of different buffer solutions: the leading one containing ions with larger mobility than the analytes, and the terminating one with ions of smaller mobility than the analytes. After the application of a high voltage, the separated ions form a sequence of zones according to their decreasing mobilities, which are detected for analytical determination.

Capillary isoelectric focusing (CIEF) separation is based on differences in the isoelectric point vales (pI) of analytes. In the gradient of pH generated in the capillary, from the lowest value at the capillary inlet to the highest at the outlet is measured. After application of a high voltage, the analytes move in the capillary until they reach an electrolyte zone where their pH are is equal to their pI.

Capillary electrochromatography (CEC) is carried out in a capillary packed with a stationary phase, where mobile phase movement is based on electroosmotic flow. The separation of analytes is based on the combined effect of the partition between their stationary and mobile phases and their electrophoretic mobility.

## 3. Application of Different Detection Methods

The analytical technique which is applied in CE systems for detection after separation step has a crucial role in the limits of detection of separated analytes obtained when no additional on-line or off-line preconcentration operations are used. The use of different analytical techniques depends on the technical conditions of carrying the separation process in a strong electric field and on the design of the detection cell with small dead volume, in order to avoid disturbances of the quality of the separation process occurring in the capillary. In the abovementioned pioneering works, potentiometric [1] and laser-induced fluorescence [2] detection were employed. The latter is simple to realize by the use of a miniature window in the separation capillary, and this detection is the most commonly offered in commercial instrumentation. Quite early in CE separations, LIF detection was applied as an especially sensitive method, and kits for such techniques are also offered by several manufacturers. Applications for electroanalytical techniques requiring the electroactivity of analytes are reported more rarely, although such detectors also can be found on the instrumental market. The use of a mass spectrometry detection in CE, which allows the identification of separated analytes, is especially powerful, and such hyphenation is also offered by highly specialized producers of CE instrumentation. Applications of other detections, such as those using atomic spectroscopy techniques, are only found practically in the laboratory. The use of each mentioned detection in a direct mode depends on the particular properties of the analyte or the possibility of the suitable derivatization or in-capillary transformation of the analyte into other product, which can be detected by a given detector.

### 3.1. Application of UV Detection

Detection based on absorption of UV radiation is the most commonly used in routine CE analytical determinations. For analytes, non-absorbing UV radiation an indirect UV detection can be used, based on the addition of the UV absorbing co-ion to BGE, which is locally replaced in the flowing stream by the analyte, resulting in a quantifiable decrease in the background absorbance. This displacement depends on the charges and mobilities of the probe and the analytes. In principle, indirect UV detection is universal, but the optimization of measuring conditions can be quite complex. The limit of detection in that mode of detection depends on the absorption coefficient of the absorbing probe in the BGE and its concentration, and too large a concentration of the probe in the BGE results in an increase of BGE conductivity. This is essential because the BGE conductivity should be smaller or comparable to the conductivity of sample in order to obtain a satisfactory efficiency of separation and prevent electro-dispersion.

The majority of inorganic ions occurring in natural waters do not absorb UV radiation. Thus, they can most commonly be determined by CE through indirect detection or in direct mode after derivatization to form products absorbing UV radiation. A variety of probes can be employed for indirect UV detection. For the detection of anions, chromate, benzoate, and phthalate are most frequently employed, while chromate and 1,2,4,5-benzene tetracarboxylic acid (pyromellitate) are the most popular choices as they give optimal peak shape and sensitivity for small anions. Pyromellitate is preferred to chromate, as chromate is potentially toxic and requires more handling care. For the CE separation of cations with indirect UV detection, imidazole and pyridine are used as additives to the BGE. The limit of detection of such methods depends on difference in absorbances between the co-ion in the BGE and the analyte, on the transfer ratio of the analyte, which is the number of equivalents of the carrier electrolyte co-ion displaced by an equivalent of the analyte ions, the ratio of charges, and the ratio of electrophoretic mobilities of the analyte and the co-ion used as a UV probe. The obtained LOD values at the mg L^−1^ level are sufficient for use in water analysis without additional preconcentration steps.

Several applications have been reported for inorganic cations with indirect UV detection in low ionic strength samples, such as tap water [17,18,19], wastewater [19], mineral water [18] and diluted high ionic strength sea water samples [20]. In indirect determinations of cations, pyridine [17], 2,4-trimethylpyridine [20], and benzimidazole [18] were recently reported as satisfactory UV probes. For the separation of inorganic cations, in order to selectively modify their similar mobility for a better resolution, the addition of complexing ligands to BGE can be effective, which was reported e.g., with the use of 18-crown-6-ether [17,18,19] or lactate [20]. For instance, CE determination with indirect UV detection was reported for the determination of NH_4_^+^, Na^+^, K^+^, Ca^2+^, Mg^2+^ and Cu^2+^ cations and Cl^−^, NO_3_^−^ and SO_4_^2−^ anions at mg L^−1^ levels in cold and hot tap waters supplied to households by installations made of copper, plastic and steel water supply pipes [17]. Due to insufficient detectability, however, the determination of ammonium, copper and sulfate was not possible.

Due to its very high salinity, and large differences in the concentration levels of different inorganic ions, the CE analysis of sea water is a much more challenging task. The development of a BGE applicable to the determination of inorganic cations in high ionic strength samples by CE with indirect UV-absorption detection has been reported very recently [20]. Several small-molecule carboxylic acids, as well as several toluidines and pyridines were tested as complexing agents and chromophoric probes, respectively. The application of a BGE containing 200 mM 2,4,6-trimethylpyridine as the chromophoric probe, 250 mM lactic acid as the complexing agent and 5% *v*/*v* methanol as an organic solvent modifier allowed for the determination of the main cations in diluted sea water. All analytes were resolved in less than 4 min with obtained concentrations of 270, 8700, 360 and 880 mg L^−1^ for K^+^, Na^+^, Ca^2+^, Mg^2+^, respectively.

Usually, the aforementioned separations are conducted in non-modified fused-silica capillaries, but in order to obtain better reproducibility of CE runs, a double capillary coating with polybrene and polyanion solutions was also employed [18]. The presented method has been successfully applied for the determination of 12 metal cations, including alkali, alkaline earth, transition metals, and ammonium in tap and mineral water samples with base-line resolution, in time periods up to 8 min. CE with indirect UV detection and PMA as UV probe were employed for the determination of sulfur-containing anions in pond water [21], while using chromium trioxide (CrO_3_) as a UV probe capillary coated with polybrene and SDS for determinations of chloride and sulfate in saline oil-field water [22]. In the determinations of phosphate and calcium, Ca^2+^ ions were complexed with 2,6-pyridine-dicarboxylic acid (2,6-PDCA) in BGE to form Ca[PDCA]_2_^2−^ complex, and then HPO_4_^2−^ and Ca[PDCA]_2_^2−^ were separated with the direct UV detection used for calcium complex detection, while indirect UV detection was employed for phosphate determination. [23].

In the process of drinking water disinfection with ozone, the formation of toxic bromate as a byproduct takes place, and it can be determined in CE systems. In one such system, isotachophoresis was combined with capillary zone electrophoresis in an automated electrophoretic analyzer with the column-coupling technique, operating in hydrodynamically closed separation system for the determination of bromate in drinking water samples [24]. The detection was carried out at 200 nm, which is selective for the determination of bromate, but not nitrate and nitrite, but other ions do not provide significant absorbance at this detection wavelength. In another example, an open-tubular capillary electrochromatography (OT-CEC) setup with UV detection was designed, where a capillary coated with trimethylamine amination polychloromethyl styrene nanolatex (TMAPL) was successfully applied for the determination of bromate in tap water [25].

CE with indirect UV detection and 2,6-pyridinedicarboxylic acid (PDC) as the UV probe was applied for fluoride determination in sea water. In optimized separation and detection conditions, an LOD of 24 μg·L^−1^ was obtained [26].

CE with UV detection was also used for the speciation analysis of various trace elements, such as mercury [27,28,29,30], selenium [31] and arsenic [32,33]. Because mercury does not exhibit UV absorption, compounds containing thiol (–SH) groups were used as chelating reagents, including l-cysteine (l-Cys) [27,29,30] and 3-mercapto-1-propane-sulfonic acid [28]. By employing UV detection, determination in real water samples became possible with the use of various pretreatment and preconcentration steps.

### 3.2. Luminescence-Based Detection Methods

Fluorescence is a very sensitive detection technique, especially when appropriate laser radiation is used as the excitation source. Laser radiation is widely used for the CE determination of numerous organic analytes, but only a few examples can be found in the literature on the determination of inorganic ions. For instance, fluorescent probes have been developed for the ultra-trace determination of heavy metal ions by CE with direct laser-induced fluorescence detection (LIF) [34]. The probes were synthetized from a macrocyclic tetraazacyclododecane-tetraacetic acid (DOTA) or diethylenetriaminepentaacetic acid (DTPA), a spacer 1-(4-aminobenzyl) and fluorescein as a fluorophore. The satisfactory separation of Ca^2+^, Mg^2+^, Cu^2+^, Zn^2+^, Ni^2+^, Co^2+^, Mn^2+^, Cd^2+^ and Pb^2+^ after derivatization was obtained using borate buffer with polybrene as the BGE. Detectability at lower ng L^−1^ levels, comparable to that obtained by CE-ICP-MS, was achieved. The application of the method for the determination in river water samples was demonstrated.

Indirect fluorescence detection using LED as a light source has been reported for the simultaneous determination of bromide, chloride, nitrate and sulfate [35]. For the fluorescent probe, 8-hydroxy-pyrene-1,3,6-trisulfonic acid (HPTS) was used in the BGE, and limits of detection of 0.4 mg L^−1^ for sulfate and 1.4 mg L^−1^ for chloride were obtained.

### 3.3. Electrochemical Detection Methods

In the search for the most reliable and universal detection methods employing CE determination of inorganic ions, electrochemical methods of detection [36] and conductivity detections play an important role, with a dominant role being played by contactless conductivity detection where the employed electrodes do not contact the BGE in the on-capillary design. Capacitively coupled contactless conductivity detection (C^4^D), in its currently used form, was invented in 1998 [37,38], and is currently commercially available. Different aspects of its use in conventional capillaries and on microchip electrophoresis devices, have been extensively reviewed by Kuban and Hauser [39,40]. With the axial and contactless configuration of electrodes positioned on the outside surface of the capillary, many advantages can be obtained, such as avoidance of the corrosion of the electrodes, prevention of electrode fouling, inherent decoupling from the electric field applied for separation, simple construction of the detector cell and the possibility of miniaturization. As the detection sensitivity is strongly dependent on background conductivity, it is recommended to select BGEs with low conductivities. In order to obtain the best limits of detection, a difference between the BGE conductivities of the analyte zones should be high, but too high a BGE conductivity can be a source of a large detector noise, while too low a BGE conductivity may cause an unwanted electro-dispersion. Conductivity detection favors the determination of poor- or non-UV absorbing charged species of relatively high specific conductivity, such as inorganic ions. The detection limits achieved for the determination of alkali and alkaline earth cations and ammonium ions with C^4^D are generally one to two orders of magnitude better than those for the indirect UV detection.

In order to obtain high separation efficiency with low conductivity and appropriate ionic strength, organic acids are used as components of BGEs, including acetic acid [41,42,43,44,45], acetic acid with histidine [8,46,47,48,49,50,51,52,53,54,55,56,57,58], 3-(*N*-morpholino)propane-sulfonic acid (MOPS) and bis(2-hydroxyethyl)amino tris(hydroxymethyl)methane (bis-tris) [59], 2-(*N*-morpholino) ethanesulfonic acid (MES) with histidine [51,60,61], pyromellitic acid (PMA) with histidine [62], and lactic acid with histidine [49,63]. For instance, the determination of selected anions has been carried out under conditions with suppressed electroosmotic flow by appropriate pH and polarity switching, with capillary coating [54,62], or by reversing the direction of the EOF by adding an EOF modifier (e.g., cetyltrimethylammonium bromide (CTAB) [61]. A low viscosity and low conductivity BGE composed of *N*,*N*-dimethylformamide (DMF) and acetic acid (1:1) was proposed for the CE-C^4^D determination of the same anions and, using such conditions, the separation of BF_4_^−^, ClO_4_^−^, PF_6_^−^ I^−^, NO_3_^−^, Br^−^ and Cl^−^ was successfully performed in 30 min, with LODs in the range of 0.83 to 3.83 μM [64].

### 3.4. Mass Spectrometry Detection

The hyphenation of CE separation to mass spectrometry (MS) detection allows for a highly sensitive and element-specific determination. This has been shown to be advantageous for a variety of applications and has been a crucial tool in the development of a new methods. While coupling CE to electrospray ionization–MS is routinely performed, for example by using a coaxial sheath-flow interface, the hyphenation of inductively coupled plasma mass spectrometry (ICP-MS) is more technically challenging. Direct detection with element specificity and multi-element measurement properties is offered by ICP-MS. In water analysis, CE-ICP-MS systems were used mostly for the speciation analysis of mercury [65,66], arsenic [67,68] and selenium [68].

The main challenge for the successful application of the CE-ICP-MS technique is the development of a suitable interface, a novel and highly efficient interface to be directly used as the nebulizer was developed for the simultaneous determination of ten arsenic species [67]. This interface was fabricated using a CE-ESI-MS sprayer kit, which was previously used to couple CE with ESI-MS. Using this proposed interface, the liquid from the capillary can be introduced directly into the sprayer chamber of the ICP-MS after nebulization. The use of this interface reduced the dead volume, narrowed the peak width and led to a higher sensitivity and a better electrophoretic resolution. Due to the detection limits in the range of 0.9–3 ng·g^−1^, the method was successfully applied in the determination of arsenic in ground waters without any sample pretreatment. The modified interface was presented by the same research group for the simultaneous separation and determination of six arsenic and five selenium species [68]. Using the same sprayer and a home-made direct-injection high-efficiency nebulizer chamber, the dead volume of the system and the dilution of analytes were decreased significantly. The method was suitable for the direct speciation of arsenic and selenium in real samples. The obtained LODs ranged from 0.11 to 0.37 μg·L^−1^ for the As compounds, and from 1.27 to 2.31 μg·L^−1^ for the Se compounds.

A short capillary electrophoresis coupled with ICP-MS was applied for the separation and determination of methylmercury and inorganic mercury Hg(II) [66]. A commercially available MicroMist nebulizer and a laboratory-made removable interface were used in the system. The developed method provided rapid separation in less than 60 s, high throughput of the samples and detectability at the μg·L^−1^ level without sample pretreatment. The CE-ICP-MS technique was also used for mercury speciation (MeHg, EtHg and Hg(II)) after complexation with mercaptoacetic acid [65]. A commercial CEi-SP20 interface system was applied to the hyphenation of ICP with CE. A successful separation was obtained in 35 min, but application to the analysis of real tap water samples required sample preconcentration. Recently, CE coupled with ICP-MS detection has been also proposed for the determination of total contents of rhenium and the speciation analysis of Re(VII) and Re(IV) in groundwater [69]. LODs of 0.02 μg·L^−1^ and 0.01 μg·L^−1^ were obtained for total rhenium and Re(VII), respectively. The quantification of Re(IV) was calculated as the difference between the total amount and the Re(VII) content.

**Figure 1:**
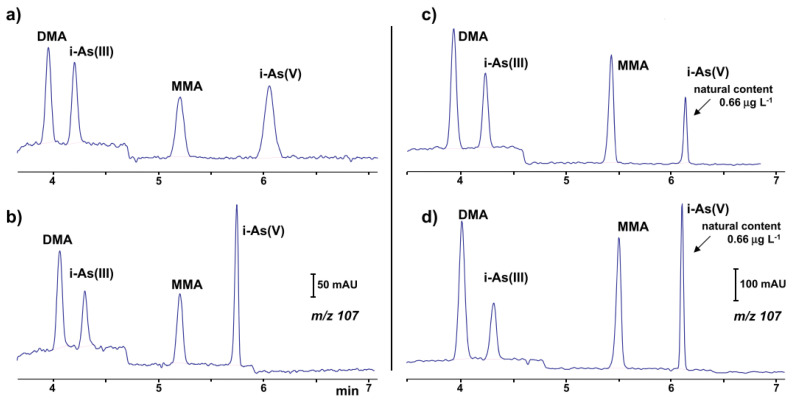
Ion electropherograms recorded in a CE-MS system for the determination of organic and inorganic arsenic compounds [70]: (**a**,**b**) 25 μg·L^−1^ standard solution (**c**,**d**) water sample with a natural content of 0.66 μg·L^−1^ i-As(V) and spiked with 1.0 μg⋅L^−1^ of DMA (dimethylarsinate), i-As(III) and MMA (mono-methylarsonate); (**b**,**d**) 10% (*v*/*v*) of a 11% (*v*/*v*) formic acid solution added to sample before injection. CE conditions: capillary—72 cm, 50 μm I.D., BGE—57 mM HFIP (hexafluoro-2-propanol) at pH 10.3, hydrodynamic injection—36 s at 50 mbar, V: +30 kV. (Reproduced under permission from Elsevier. License Number 5173120352687).

The identification and simultaneous quantification of organic and inorganic arsenic (mono-methylarsonate (MMA), dimethylarsinate (DMA), As(III) and As(V)) species at low concentration levels using the CE-ESI-MS setup [70] was recently reported. The coaxial sheath liquid sprayer was used for the CE-MS coupling. Using hexafluoro-2-propanol as the BGE increased the separation efficiency, but the detection of these compounds by ESI-MS was not as sensitive as the detection obtained by ICP-MS. Therefore a preconcentration step was necessary for the analysis of real water samples. Limits of detection between 0.02 and 0.04 μg·L^−1^ were found using partial evaporation of the water sample (50:1). The proposed method was used in the analysis of groundwater and bottled water (Figure 1).

In much earlier work, electrospray tandem mass spectroscopy (ESI-MS/MS) with multiple reaction monitoring (MRM) was optimized for the detection of As(III) and As(V), selenium (Se(IV) and Se(VI)) and bromate with on-line enrichment of the target analytes [71]. The LODs obtained were in the range of 1–3 ng·mL^−1^. ESI-MS detection was also demonstrated for the determination of four chlorine species (ClO_3_^−^, ClO_2_^−^, ClO_4_^−^, Cl^−^), with LODs at the mg·L^−1^ level achieved [72].

### 3.5. Atomic Spectrometry Detection

For the speciation of metal ions, an attractive possibility is CE coupling to atomic absorption spectrometry instruments. The CE separation in a setup using electrothermal atomic absorption spectrometry (ETAAS) has been demonstrated, for instance, for selenium speciation [73]. In a developed interface, the introduction of the CE effluent directly through the end of the graphite tube was used, where the elimination of the upper injection hole of the graphite tube reduced the loss of the analyte and enhanced detection sensitivity. In combination with solid phase extraction (SPE), limits of detection (LOD) values of 0.18, 0.17, 0.54, 0.49 μg·L^−1^ have been reported for the determination of Se(VI), Se(IV), SeMet, and SeCys_2_, respectively.

In the case of hydride-forming analytes, the application of a CE–HG–ETAAS setup provides improvement of the detection limits by 1 to 3 orders of magnitude. A designed interface offers good stability, good gas–liquid separation efficiency, a smaller dead volume, good reproducibility and easy operation for As(III) and As(V) determination [74]. The detection limits of As(III) and As(V) were 135 ng·g^−1^ and 160 ng·g^−1^, respectively.

Detailed information about applications of CE systems with various detections in water analysis published in recent decades are presented in Table 1.

## 4. Sample Processing in CE Systems for Water Analysis

The determination of numerous environmentally important anions and cations in natural or processed waters which are present in trace concentration levels requires suitable limits of detection and sufficient selectivity of the developed methods. Due to the dimensions of capillaries used, in order to obtain a satisfactory resolution only a very limited sample volume can be introduced, which may usually affect the obtained detectability. An additional factor that has to be considered in case of natural samples in designing the sample processing steps is the matrix of the natural media. Although some water samples do not require preliminary processing besides dilution or filtration, in many cases an insufficient LOD and heavy loaded matrix do require some preliminary processing. In CE techniques, this can be performed using conventional off-line procedures, but in capillary electrophoresis it is quite convenient to carry out them on-line in especially designed CE setups. Particularly attractive are the procedures that can be carried out on-line or, even better, in the in-capillary mode, or directly in the separation capillary. Each of those methods has its limitations and advantages.

### 4.1. Off-Line Sample Processing

There are numerous developed sample pretreatment procedures and methods employed in trace analytical techniques, which can be used with unlimited sample volume. The most frequently used are liquid–liquid extraction (LLE) and solid–phase extraction (SPE) methods in their different technical modifications. The usual drawbacks of such procedures are the time needed to perform them, limited precision and also the possibility of the contamination. For instance, SPE with 5-sulfosalicylic acid (SSA)-functionalized silica-coated magnetic nanoparticles (SMNPs), was used for the extraction of selenium species [73]. The enrichment factors for Se(VI), Se(IV), SeMet, and SeCys_2_ were 21, 29, 18, and 12, respectively. In another example of off-line pretreatment for arsenic speciation, the possibility of carrying out a preconcentration by partial evaporation of the solvent 50:1 from the water sample at 55 °C was reported [70]. The limits of detection were found to be between 0.02 and 0.04 μg·L^−1^ in a CE-ESI-MS system. The recovery of the analytes during the evaporation was in the range of 76 to 108%.

Both LLE and SPE can be carried in a microscale, as microextraction techniques seems to be more suitable due to a use of significantly lower volume of the organic phase. In the liquid-phase microextraction (LPME) procedure, the analytes from an aqueous sample solution are extracted into a small volume (μL) of organic solvent, which usually eliminates the interfering matrix components. Especially for CE, the use of these techniques is beneficial, but conversion of organic acceptor into an aqueous solution is usually required because aqueous solutions are more suitable for direct injection to CE. For solid-phase micro-extraction (SPME) porous fibers are used for the adsorption of analytes.

One recently-accepted technique is extraction into supported liquid membranes (SLMs), which are usually made of a porous inert material with an immobilized organic solvent; analytes are transferred across the SLM from donor solution into the acceptor due to diffusion or the electric field. In additional electromembrane extraction (EME) carried out in the electric field, a shorter extraction time than that of SLM extraction can be used, and whole process can be more selective [75]. In order to develop an EME procedure for particular determination, the SLM composition, the extraction voltage, pH of the sample and acceptor solutions, extraction time, agitation and temperature must be optimized. Numerous EME procedures have been developed for applications with the LC, GC and CE methods [76].

As far as CE systems are concerned, EME preconcentration was used in the CE determination of metal ions (Mn^2+^, Cd^2+^, Zn^2+^, Co^2+^, Pb^2+^, Cu^2+^, Ni^2+^), to name one example [46]. The limits of detection obtained in the EME-CE-C^4^D determination ranged from 25 to 200 nM, which is one to two orders of magnitude improved, compared to CE-C^4^D without sample treatment. A similar set-up was used for metal determination in diluted saline samples, however due to substantial matrix effects, the obtained sensitivity was worse (500 nM) [41]. An EME with a porous polypropylene hollow fiber impregnated with 1-heptanol was applied for perchlorate extraction from drinking water [53]. Sub-ppb concentrations of perchlorate can be detected in natural water samples, demonstrating that such a method is suitable for the determination of perchlorate below the United States Environmental Protection Agency (US EPA) limit. In the CE-C^4^D system, EME was also employed for the purification and enrichment of bromate in drinking water [45]. Bromate could be preconcentrated up to 267-fold. For the simultaneous extraction of inorganic and organic mercury species with high efficiency prior to speciation analysis by capillary electrophoresis, the hollow fiber-supported liquid–liquid–liquid membrane microextraction method was reported [30]. For a 50 mL sample solution, the obtained enrichment factor values were 103, 265, 511 and 683 for Hg^2+^, MeHg, EtHg and PhHg, respectively.

In a micro-electromembrane extraction system, free liquid membranes (FLMs) were reported as being possible to use, being formed in a narrow bore tube as the plug of a water-immiscible organic solvent sandwiched between two plugs of aqueous solutions (donor and acceptor) [43]. This technique was demonstrated in the determination of trace concentrations of perchlorate, where an enrichment factor of up to 30 for perchlorate was achieved, and it made use of the CE-C^4^D system possible. Organic solvent consumption in extractions across FLMs is reduced compared to SLMs.

**Figure 2:**
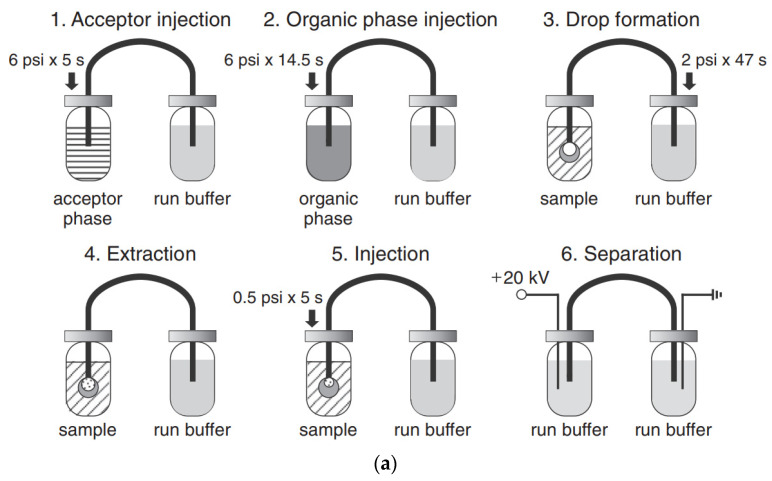

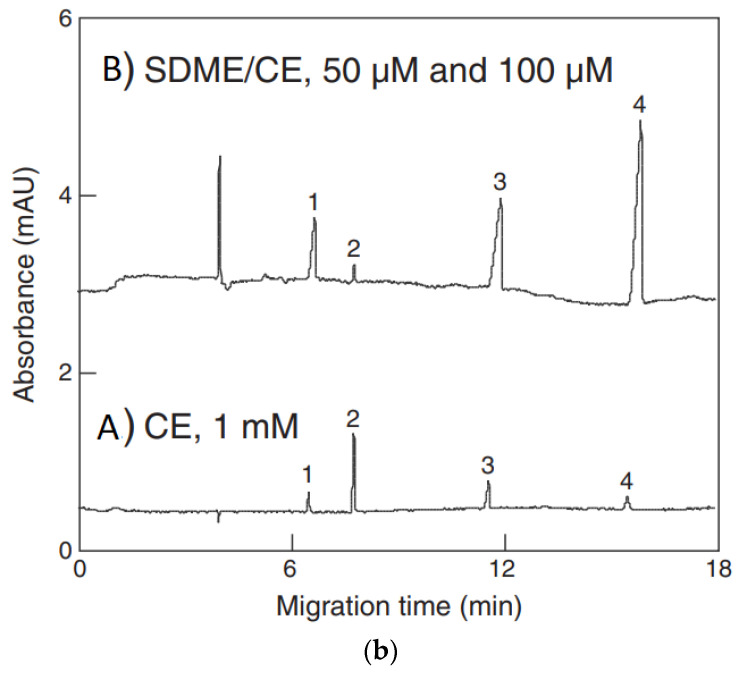
Application of a single drop microextraction (SDME) procedure (**a**) for inorganic arsenic species in water by CE and recorded example electropherograms (**b**) [32]. In (**a**): (1) acceptor injection, (2) organic injection (trioctylmethylammonium chloride, aliquat 336 in octanol), (3) drop formation, (4) extraction, (5) sample injection, and (6) separation. In (**b**): (**A**) CE of 1 mM arsenic compounds, (**B**) SDME/CE of 100 μM of DMA and As(III) and 50 μM MMA and As(V) in unbuffered water (5 min SDME). Arsenic compounds: DMA (1), As(III) (2), MMA (3), and As(V) (4). CE conditions: capillary—60 cm (50 cm), 25 μm I.D., BGE—15 mM phosphate buffer at pH 10.6, V: +20 kV, UV detection at 200 nm, hydrodynamic injection for 5 s at 0.5 psi. (Reproduced under permission from Elsevier. License Number 5173111119111).

The use of pretreatment systems mechanically integrated with CE setups is usually carried out in prototype, laboratory-made devices, which are not commercially available. For instance, a promising device for the efficient preconcentration of a single-drop micro-extraction (SDME) coupled with in-line CE commercial instruments has been presented [77,78]. Analytes are extracted from a sample donor solution into a drop of acceptor solution hanging at the inlet tip of a capillary. The enriched drop is then introduced into the capillary for CE analysis. The acceptor solution and next organic phase are sequentially injected into capillary filled with BGE, then using a backpressure, a drop of the acceptor phase covered with a thin layer of the organic phase is created on the capillary inlet and immersed in sample solution. Finally, the enriched acceptor phase is injected into the capillary. Due to the large volume ratio between the sample donor phase and the acceptor drop as well as the thin organic layer, high enrichment factors can be obtained with SDME in a short time. SDME-CE-UV methods were applied, for instance in arsenic speciation [32]. The preconcentration procedure is schematically shown in Figure 2. The enrichment factors obtained with extraction times of 15 min were 390, 340, 1100, and 1300 for As(III), DMA, MMA, and As(V), respectively. The limits of detection with UV detection were 0.2, 0.7, 0.1, and 0.2 μM for As(III), DMA, MMA, and As(V), respectively.

**Figure 3:**
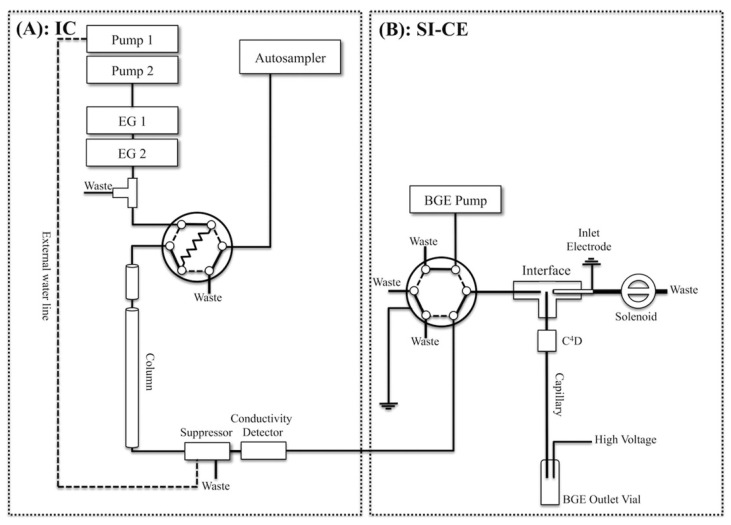
Schematic diagram of a two-dimensional ion chromatography (IC) (**A**) and sequential injection (SI)-capillary electrophoresis (CE) system with conductivity detections developed for the determination of inorganic anions and organic acids in waters (**B**) [79]. (Reproduced under permission from ACS, licence is not required).

One kind of instrumental integration was demonstrated by the design of an online two-dimensional ion chromatography (IC)-CE system (Figure 3), developed for the determination of major inorganic anions, i.e., bromate and organic acids, in tap water [79]. A six-port, two-position injection valve in the sequential injection (SI)-CE setup and a T-piece interface were used for coupling. A two-dimensional IC effluent was sequentially and electrokinetically injected into the CE with a sampling time of 18.4 s. The system was tested for the determination of anions typical for the drinking water matrix and drinking water disinfection by-products. A 10-fold increase in peak capacity compared to IC was obtained due to the orthogonality of the separation mechanisms of those two methods.

### 4.2. On-Line (In-Capillary) Pretreatment Operations

Simpler procedures, in terms of their technical development, seem to be operations performed in the separation capillary by appropriate changes to the electric field and the sequence and kind of introduced solutions. The optimization of such procedures is, admittedly, very tedious, because of the complexity of the physico-chemical phenomena taking place during such operations.

An increase to the volume of the sample introduced into capillary results in worsening of the separation efficiency. Certain methods of the introduction of larger amounts of analytes from the sample to be analyzed without deterioration of the resolution, with simultaneous preconcentration of analyte(s), have been developed. For this purpose, a stacking effect can be employed by combining capillary zone electrophoresis with isotachophoresis. In both cases, the electromigration phenomenon in the capillary under applied high voltage is utilized. The preconcentration of the analyte as effect of stacking is based on its concentration in narrow segments of the interphase, separating zones of weak and strong electric fields so the stacking effect can be created by introducing a sample solution of much lower conductivity than that one used in BGE. For example, the field amplified sample stacking technique for on-line sample preconcentration was applied in the analysis of five sulfur-containing anions, and the LOD values (from 0.02 to 0.12 μg mL^−1^) were improved by about 4-fold [21]. The CE runs were performed by introducing a water plug prior to the electrokinetic injection of the sample. The same technique was also employed in trace bromate enrichment using OT-CEC as a separation method, with a 10-fold improvement of the LOD [25].

A pressure-assisted electrokinetic injection (PAEKI) has been used for the on-line pre-concentration of arsenic (As(III) and As(V)), selenium (Se(IV) and Se(VI)) and bromate (BrO_3_^−^) [71]. This technique utilizes the principles of both counter-current electro-concentration and stacking. During the electrokinetic injection of the sample, the velocity of the electroosmotic flow is balanced to an external hydrodynamic pressure to create a stationary boundary at the inlet of the capillary where the target analytes accumulate, according to the principles of stacking. For on-line enrichment coupled with CE-ESI-MS/MS, sensitivity LODs of 1–3 ng·mL^−1^ were achieved, which were below the maximum contaminant levels in drinking water for all five anions studied. This technique seems to be very promising, as the enhancement depends on the sampling time only, and it is not limited by the capillary dimensions.

**Figure 4:**
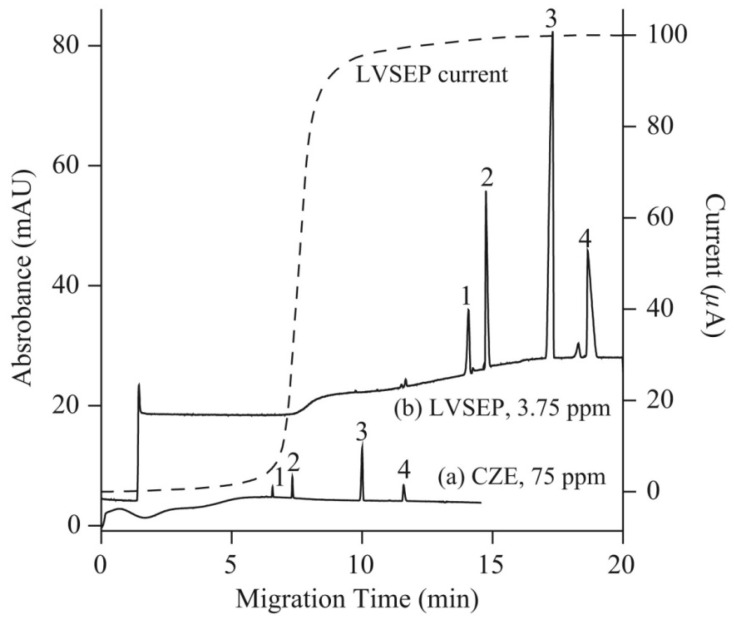
Electropherograms recorded for arsenic speciation using CE-UV without (a) and with (b) large volume sample stacking with an electroosmotic flow pump (LVSEP) (b) [33]. In (a): recording for 75 ppm As of each As species (1; As(V), 2; MMA, 3; As(III), 4; DMA) in deionized water injected at 0.5 psi for 5 s, and (b): recording LVSEP of 3.75 ppm of each As species in 0.1 mM sodium phosphate buffer with full injection. CE conditions: μSiL-FC coated capillary—60 (50) cm, 50 μm I.D., BGE—100 mM sodium phosphate at pH 9.6, V: 20 kV, UV detection at 200 nm. Dotted line: electric current. (Reproduced under permission from Elsevier. License Number 5173120740442).

Several on-line sample preconcentration techniques, such as large volume sample stacking with an electroosmotic flow pump (LVSEP), field amplified sample injection (FASI), transient isotachophoresis (*t*ITP), and electrokinetic supercharging (EKS) combining FASI and *t*ITP, and counter flow CF-EKS, were compared to improve the sensitivity of detection of arsenic speciation in CE-UV analysis [33]. For separation, a cross-linking, fluorocarbon polymer-coated fused silica capillary (μSiL-FC) was used to reduce the EOF with a buffer of a high ionic strength. Figure 4 presents electropherograms of arsenic species obtained without preconcentration, with full injection and removal of the sample matrix (LVSEP). Enrichment in the range of 130–180-fold was possible using this procedure. The best results of the enrichment were achieved using the CF-EKS procedure. A larger quantity of the sample was injected during the FASI process due to sample zone movement, which was exactly counter-balanced by a counter flow, using pressure. Enrichment factors of 45,000, 18,000, 6300 and 7100 were obtained for the standards of As(V), MMA, As(III) and DMA respectively. For natural water samples, due to the presence of complex matrix components, the enrichment factors decreased, but nevertheless the limits of detection obtained with UV absorbance detection were in the range of 2–9 ppb of As.

An on-line column-coupled capillary isotachophoresis–capillary zone electrophoresis (ITP-CZE) automated analyzer was reported upon for bromate determination with the use of an isotachophoretic separation step, and sufficient resolutions of bromate from the macro-constituents, i.e., sulfate, chloride and nitrate, as well as from the micro-constituents, i.e., nitrite, fluoride, phosphate were obtained [24]. A leading type of *t*ITP–CZE method was applied for fluoride determination in seawater [26]. Chloride acted as the leading ion, and 2,6-pyridinedicarboxylic acid (PDA) in BGE as the terminating ion. In optimized separation conditions and using indirect UV detection, an LOD of 24 μg·L^−1^ was achieved.

One can also find in the literature examples of a combination of off-line preconcen-tration processes with an on-line stacking strategy employed for further improvement of the detection limits, for trace analysis by CE. For instance, an SPE with nanometer-sized Al_2_O_3_ as a sorbent, together with field-amplified sample stacking, was used to preconcentrate inorganic selenium species [31]. The enrichment factors related to two preconcentration steps were 41367-fold and 61935-fold for Se(IV) and Se(VI), respectively. By the combination of off-line SPE and on-line stacking, the LODs of the developed methods were greatly improved, namely to 57 and 71 ng·L^−1^ for Se(IV) and Se(VI), respectively. However, the processing time of the whole method was rather long, due to the time-consuming off-line SPE step (over 80 min per sample).

In a CE-ICP-MS system, a stacking step was carried out together with dispersive solid–phase extraction (DSPE), which was successfully applied in the determination of ultra-trace levels of MeHg, EtHg and Hg(II) [65]. Thiol cotton particles pretreated with mercapto-acetic acid were used as the solid phase in the off-line method for preconcentration. The stacking step enhanced the sensitivity of the CE-ICP-MS 25-fold, 29-fold and 27-fold for MeHg, EtHg and Hg(II), respectively. Using both methods of preconcentration, ultra-high sensitivity was achieved with LODs in the range of 9–11 ng·L^−1^. In another work dealing with mercury speciation, a different sample preconcentration technique was also used, namely phase transfer-based liquid–liquid–liquid microextraction (PT-LLLME), together with large volume sample stacking (LVSS) [29]. LVSS with polar switching was performed for the matrix removal. Under optimized conditions, enrichment factor values ranging from 160 to 478 were obtained for the extraction step of the target mercury species. By combining PT/MS-LLLME with LVSS-CE/UV, the enrichment factor values were magnified from 2250 to 12,138, and the limits of detection were obtained at the ppb level (1.40–5.21 μg·L^−1^).

## 5. Simultaneous Determination of Anions and Cations

One attractive feature of capillary electrophoresis in water analysis applications is the possibility of simultaneous determinations of anionic and cationic species. Such a possibility is provided to some extent by multicomponent techniques of atomic emission spectroscopy, but with a single signal for the total content of all species containing particular element. Such determinations can also be carried out by the appropriate configurations of ion chromatography setups, such as in a non-suppressed mode of IC connecting anion-exchange and cation-exchange columns [80], by a two dimensional ion chromatography using one-valve switching and two columns [81], or in dual-capillary ion chromatography system [82].

Simultaneous CE determinations can be carried out in dual capillary systems, but a unique feature of the CE technique using appropriate measuring methodology is the possibility of the simultaneous determination of anions and cations in one separation capillary in a single run. This is possible with the application of a large electroosmotic flow and a normal mode of polarization, when cations migrate to the detector together with EOF, when the EOF is larger than the mobilities of anions, and when anions migrate to the detector. It has to be admitted, however, that due to the large mobility of inorganic ions, satisfactory separation under such conditions is difficult to optimize, and different strategies to cope with this difficulty have been reported, see e.g., review [83]. Some attempts reported so far were based on a special injection method. For instance, the dual opposite-end hydrodynamic injection of the sample was proposed for simultaneous inorganic ions in drinking water [62]. Two sample plugs were placed in the two opposite ends of the capillary prior to the separation. Due to the migration of cations and anions in opposite directions, the optimization of the C^4^D detector position along the capillary was necessary in order to achieve a satisfactory separation of ions. Using PVA-coated fused silica capillary of 60 cm length, 11 cations and anions were separated in less than 3 min with LODs in the range 0.07–2 mg·L^−1^.

**Figure 5:**
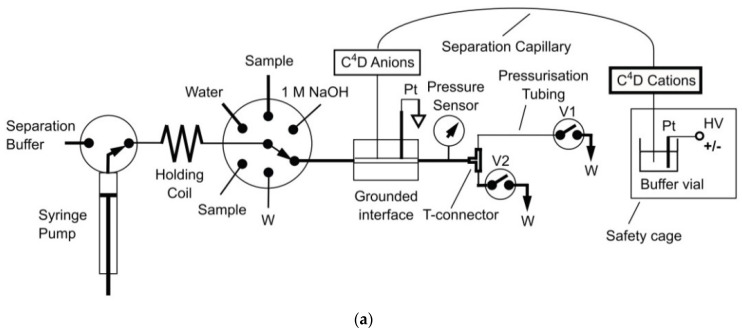

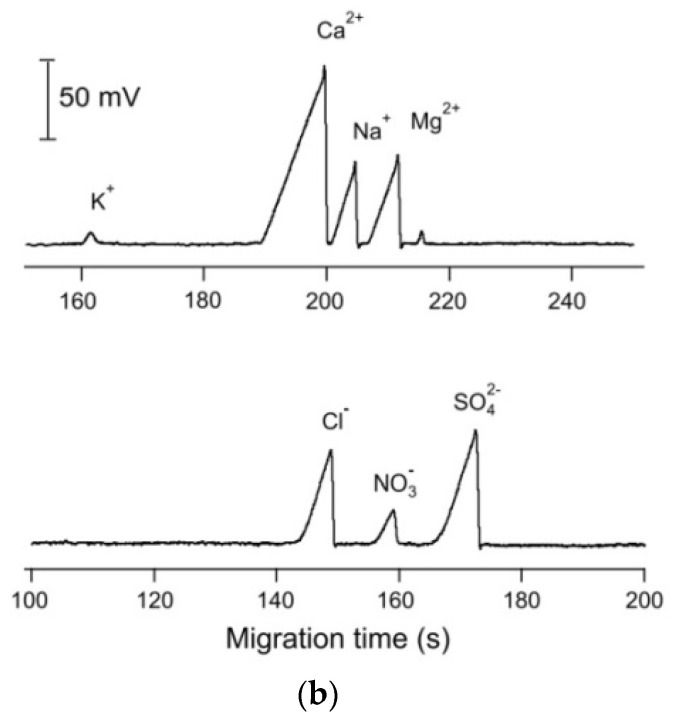
Schematic diagram of an SIA-CE system with dual contactless C^4^D detectors for simultaneous determination of anions and cations (**a**), and an example of its application in the analysis of tap water (**b**) [58]. In A: C^4^D: contactless conductivity detector; HV: high-voltage power supply; W: waste; and V1, V2: stop valves. In B: tap water of pH 4 was filtered and diluted 5 times before injections. Both sample plugs were injected at the grounded end of the capillary, with the plug of the anion sample delivered to the HV-end of the capillary with a pressure of 4.5 bar within 90 s. CE conditions: capillary—50 cm, 10 μm I.D. (*L*_eff_ for cations—43 cm and *L*_eff_ for anions—35 cm), BGE—12 mM His, 2 mM 18-crown-6, pH 4, *E* = 400 V/cm. (Reproduced under permission from Elsevier. License Number 5173120143146).

One very helpful methos of manipulations with sample processing, injections and mechanization of whole analytical procedure is the hyphenation of CE setups to flow injection systems, see e.g., review [84]. For instance, in the application of sequential injection analysis (SIA) systems combined with CE setups, the flexible manipulation of the sample plug is possible, leading to the ability to precisely control the pressure for hydrodynamic injection, automated flushing of the separation capillary, and two-way pumping. Use of the SIA makes it possible to change the measurement conditions during the run to optimize the separation. SIA-CE was reported with different detection techniques, but in inorganic ion determination C^4^D is predominant. For instance, for the determination of inorganic ions in creek water, the SIA-CE system was employed with a T-junction as the interface and a C^4^D detector with two cells [48]. All operations of the procedure were automatically controlled, the same BGE was used for all ions, and the cations and anions were determined sequentially by changing the polarity of the separation voltage. The same authors also developed other configurations of SIA-CE systems with one or two C^4^D detectors for the simultaneous separation of major inorganic cations and anions [58]. For the analysis of tap water, dual single-end injection CE in one capillary with two C^4^D detectors was employed. Both samples were injected at the same end of the capillary, but one sample plug was delivered to the other end of the capillary by the use of pressure. Baseline separations were achieved for 9 cations and 5 anions in a single run of 4 min, with LODs in the 1.5–2.0 μM range for the anions and in the 0.3–1.5 μM range for the cations. In Figure 5, a schematic diagram of this SIA-CE system and electropherograms of tap water samples are presented.

**Figure 6:**
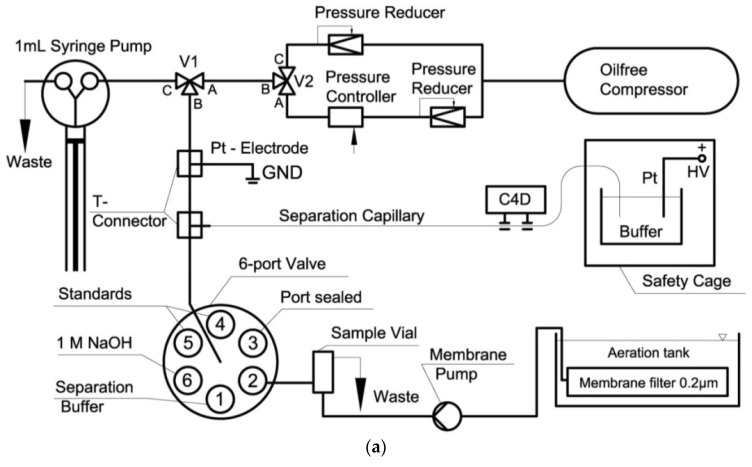

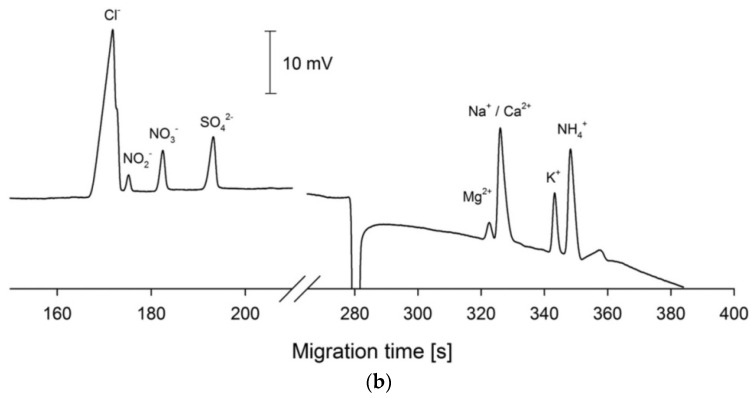
Schematic diagram of a SI-CE system with contactless C^4^D conductivity detection developed for the determination of nitrogen-containing ions (**a**), and sample electropherograms recorded from wastewater samples (**b**) [61]. CE conditions in (**b**): capillary—68 cm, 20 μm I.D., BGE—100 mM His, 100 mM MES, 0.13 mM CTAB, 1.5 mM 18-crown-6, pH 6, V: +24 kV. (Reproduced under permission from Elsevier. License Number 5173120520906).

Through detailed optimization of the SIA-CE setup with C^4^D detection (capillary length, pressure levels and separation time), it was possible to achieve the separation of inorganic cations and anions in wastewater in one run (Figure 6) [61]. The EOF was reversed by adding CTAB to the BGE, and the anion separation was performed using negative polarization. In order to move the cations towards the detector and prevent them from exiting the capillary at the injection end, a hydrodynamic flow towards the detector end was superimposed.

In the capillary filling method, the separation capillary is filled with a sample containing anions and cations dissolved in BGE, and after a high voltage application, the anions and cations move to the appropriate electrode, based on their mobilities under conditions with suppressed EOF [85]. The analytes form boundaries and migrate towards the C^4^D detector. Using the capillary with an effective length 20 cm for cations and 15 cm for anions, 8 ions (Cl^−^, NO_3_^−^, SO_4_^2−^ and Na^+^, K^+^, NH_4_^+^, Ca^2+^, Mg^2+^) were successfully separated and detected at ppm levels within 100 s in water samples. In another attempt, the separation of cations and anions was achieved by gradient elution moving boundary electrophoresis (GEMBE) [57]. GEMBE is a technique that involves electrophoretic separation in a short-length capillary using continuous injection against a variable counter-flow, which is a combination of the EOF and a pressure-controlled, variable hydrodynamic flow. Each analyte is introduced sequentially in order of decreasing electrophoretic mobility because the pressure-driven flow is reduced over time and the resolution is controlled by the pressure gradient. Seven anionic and cationic analytes were separated using non-linear gradients in less than 7 min, with LOD values in the low μM to sub-μM range.

The analytical literature provides numerous examples of dual-channel CE systems developed for the simultaneous determination of anions and cations with two capillaries [44,54,55,56]. A system with simultaneous hydrodynamic injection into both capillaries was reported, and the application of such a system was reported for the monitoring of ammonia, nitrite and nitrate during the denitrification of sewage in an activated sludge reactor [55]. Another dual-channel CE system was developed with electrokinetic injection for the separation of anions and cations in mineral fertilizer solution samples [44]. As the amount of injected sample in electrokinetic injection is highly dependent on the conductivity of each sample, careful optimization of measuring conditions is needed. Due to short capillaries (effective length 8 cm), base-line separation of all ions (NH_4_^+^, K^+^, Ca^2+^, Mg^2+^, Sr^2+^, Ba^2+^, Cl^−^, NO_3_^−^, SO_4_^2−^, ClO_3_^−^ and F^−^) was achieved in less than 1 min.

In a purpose-made, automated SIA-CE setup with two capillaries and two C^4^D detectors, a sample was simultaneously injected electrokinetically onto two separate capillaries for independent separation [54]. In order to improve the separation, coated capillaries (35 cm effective length for the cations and 28 cm for the anions) were used. Parallel separation of 11 anions and 12 cations was achieved, in a total analysis time of 3.5 min. Detailed information about applications of CE for the simultaneous determination of inorganic anions and cations in water samples are shown in Table 2.

The development of new designs and applications of microfluidic devices in recent decades is one of the most intensive directions of scientific research in analytical chemistry [86]. They are being developed both for fast single and multicomponent determinations based on CE separations [87], including programmable and reconfigurable chips [88] and paper-based microsystems [89]. Most commonly, they are designed for medical diagnostic needs, see e.g., [90], but they are also designed for environmental monitoring [91,92], including water analysis [93,94,95]. This pronounced development trend has resulted in thousands of papers now being published annually (more than 8000 papers per year in 2019–2020).

Microchip capillary electrophoresis (MCE) offers some advantages over conventional, larger scale capillary electrophoresis techniques, including the integration of different separation functions onto the chip, the consumption of smaller amounts of samples and reagents and shorter analysis time, as separations within a minute or few seconds have been reported [95]. It has to be emphasized, however, that the method of sample introduction, complexity of the sample matrix, and need for determinations of low-concentration of analytes are still crucial challenges in the use of a microfluidic devices for environmental analysis, hence their acceptance in routine laboratories is still limited.

So far, only a few applications have been presented for determinations of inorganic ions in water samples. Obviously, the most challenging task is to develop sufficiently sensitive detection devices which are compatible with the size of the miniaturized separation units. Although different detection methods can be used in microfluidic devices, the detectors successfully used for the determination of inorganic ions in waters were most commonly conductivity detectors in the contact [96,97,98,99] or contactless modes [100,101,102], mostly because of their simplicity of fabrication and the possibility of direct incorporation onto a chip. Capacitively-coupled contactless conductivity detection (C^4^D) is the most widely used and commercially available method.

**Figure 7:**
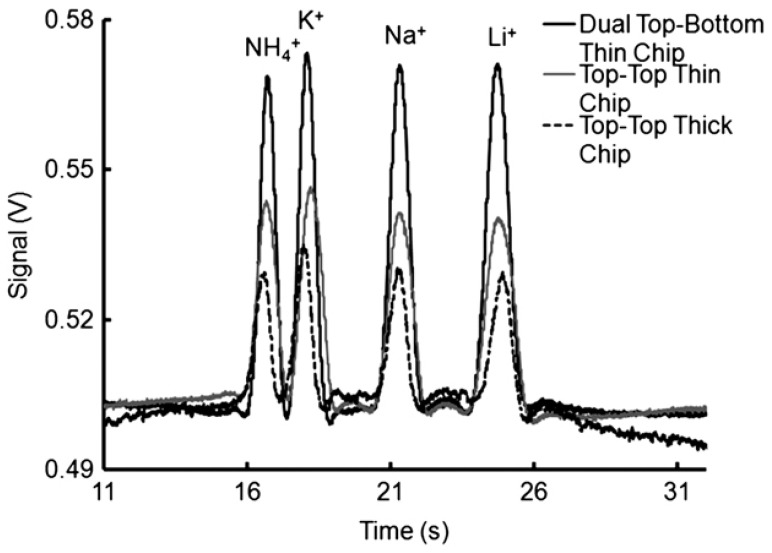
Comparison of the applications of different detector cell designs in CEs of inorganic cations with contactless C^4^D conductivity detection [101]. Electropherograms recorded for 0.5 mM cations (K^+^, Ca^+^, Mg^+^, Li^+^) using electrodes in dual top–bottom (black line), top–top (gray line) positions using thin chips, and in the conventional top–top configuration with thick chips (dotted line). CE conditions: channel—85 (65) mm × 50 × 50 μm, BGE—20 mM MES/His, 2 mM 18-crown-6, pH 6.4, injection at 0.5 kV for 5 s, V: +3 kV. C^4^D detector: a sinusoidal excitation signal of 22 *V*_pp_ (peak-to-peak) at 300 kHz; electrode distance, 1 mm; electrode width, 1 mm. (Reproduced under permission from John Wiley and Sons. License Number 5173110627787).

The MCE systems for inorganic anions offer sensitivity at the μM level. For instance, in a developed dual ‘top–bottom’ C^4^D cell configuration used with a chip made from thin plastic sheets of 125 μm (Figure 7), LOD values of 0.3 and 0.15 μM were obtained for the determination of cations and anions, respectively, in drinking water samples [101]. A novel concept of contactless conductivity detection based on an indium tin oxide-coated poly(ethylene terephthalate) film was also reported for the detection of metal ions, to enhance the efficiency of prototyping and fabricating a desired microchip [98]. The application of amperometric detection in a microchip CE was reported with the use of either graphite pencil leads [103], or screen-printed carbon-based electrodes [104] as the working electrodes. In both cases, the determination of nitrite in well water with an LOD 2.8 μM [103], and in drinking water [104] was reported. Very recently, LIF detection was employed in microchip electrophoresis for the simultaneous trace determination of silver and mercury ions [104]. The developed analytical procedure also involved an off-line preconcentration step with the use of a stirring bar modified with encoded hairpin DNA probes for the specific extraction of analytes.

Most CE microchips have been laboratory-made from polymer materials, and poly(di-methylsiloxane) (PDMS) [96,98,100] and poly(methyl methacrylate) (PMMA) [97,99,101] are the two most commonly used. For the same purpose, commercially available borosilicate glass chips can be used [102].

MCEs have been applied for the determination of anions in drinking water [96,97,104] and river water [100,102], as well as for the determination of heavy metals cations in river water [98], or alkali and alkaline earth metals in drinking water [101]. The ITP-CZE column-coupled microchip was used for NH_4_^+^ determination in waste water [99]. The sample was preconcentrated and cleaned up in the isotachophoretic capillary, and in the next capillary the cations were separated. Determination of trace ammonium in the presence of other major cations up to 400 to 800 times higher concentrations was possible. Detailed information about the applications of CE microfluidics in water analysis are listed in Table 3.

The development of robust, portable analytical measuring systems for different applications, including environmental monitoring is currently a very remarkable trend thanks to recent progress in micromechanics, material science and electronics. Portability allows analyses to be carried out outside of the laboratory, preventing or minimizing the risk of contaminating the sample and leading to faster response times at a lower cost. Portable instruments should, therefore, be lightweight and have small dimensions; they should be easy to transport, and should have independent power sources [106]. Recent trends in the design of portable capillary and microchip electrophoretic systems for field analysis have been presented in several reviews [16,95,107].

**Figure 8:**
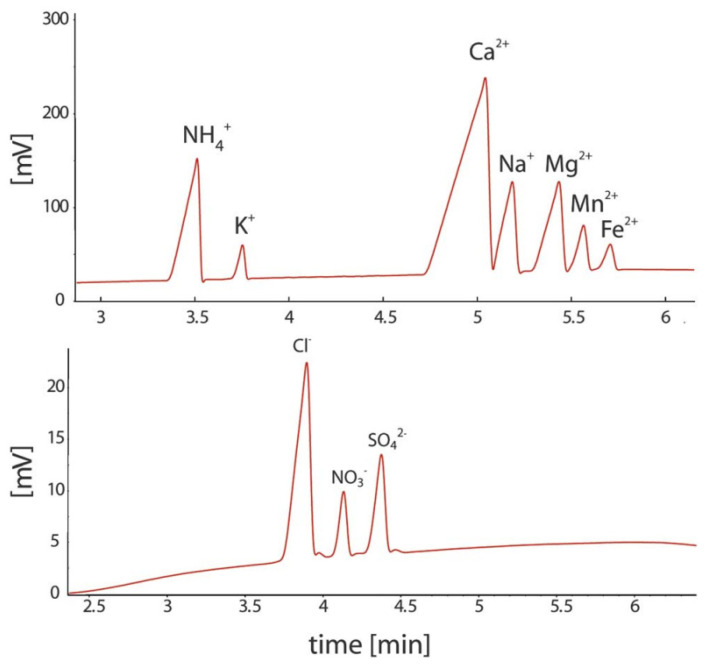
Electropherograms recorded for the CE determination of anions and cations in sediment pore water using a portable CE system with contactless C^4^D conductivity detection and hydrodynamic injection [52]. Sample: 700 μM NH_4_^+^, 100 μM K^+^, 1500 μM Ca^2+^, 500 μM Na^+^, 320 μM Mg^2+^, 120 μM Mn^2+^, and 70 μM Fe^2+^ for the cations and 610 μM Cl^−^, 120 μM NO_3_^−^, and μM SO_4_^2^^−^ for the anions. CE conditions: capillary—55 cm, 50 μm I.D., BGE—11 mM His, 50 mM AcOH, 1.5 mM 18-crown-6, 0.1 citric acid, V: ±15 kV. (Permission from RSC is not required).

Based on the designs reported so far for such systems, conductivity detection is well suited to portable CE systems for the determination of ionic species because of their straightforward miniaturization and very limited electrical power consumption. A portable CE instrument with a C^4^D detector was successfully applied in the investigation of sediment porewaters [8,52]. The separation and quantitative determination of major inorganic anions and cations, including Mn(II) and Fe(II) ions, was achieved in less than 15 min. The sediment samples were collected from lakes and analyzed immediately in the field. By avoiding the transport of the sample, its composition was preserved, which made it possible to study geochemical processes. The electropherograms of the sediment porewater samples are shown in Figure 8 [52].

**Figure 9:**
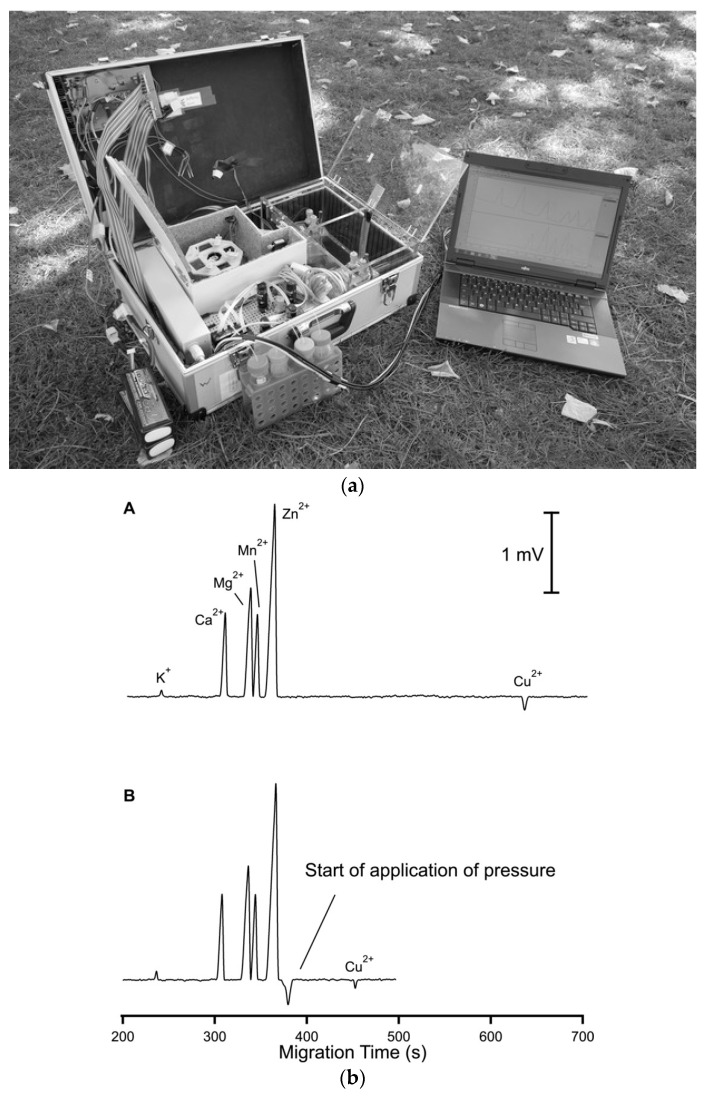
Photograph of a thermostatted dual-channel portable capillary system with contactless C^4^D conductivity detection for the concurrent determination of anions and cations (**a**) and sample electropherogram recorded for 1:100 diluted mining pond water (**b**) [63]. In a: the syringe pump and valves are in the front, the thermostatted compartment is in the center and the high voltage compartments are to the right; all compartments have been opened. In b: ions separation (**A**) without and (**B**) with pressure assistance. CE conditions: capillary—80 (65) cm, 25 μm I.D., BGE—9 mM His, 4.6 mM lactate, 25 mM acetic acid, and 1 mM 18-crown-6 ether, V: −25 kV, injection—50 kPa. Co-flow: 70 kPa applied after 375 s. (Reproduced under permission from John Wiley and Sons. License number 5173110276810).

The same research group also proposed a dual-capillary portable instrument for the simultaneous determination of inorganic anions and cations in similar samples [63]. The instrument has a compact design, with dimensions of 52 × 34 × 18 cm, and a weight of less than 15 kg. Each channel has a separate buffer container to allow for the independent optimization of separation conditions. The system is controlled by a computer and works automatically. The electropherograms of a sample of mining pond water are shown in Figure 9. Because the migration time for the copper ion was relatively long compared to the other analytes, pressure assistance was applied to shorten the analysis time.

**Figure 10:**
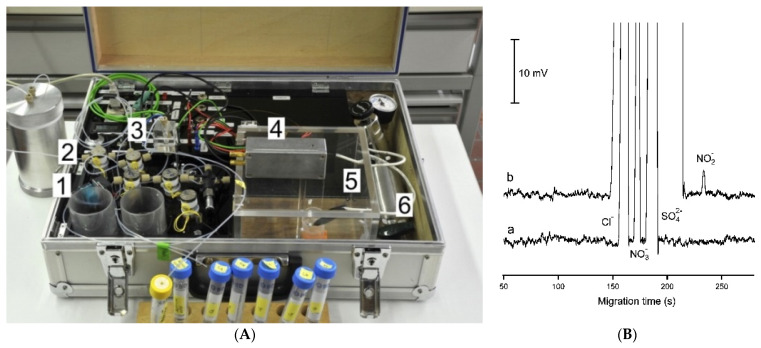
Photograph (**A**), and sample recording of an electropherogram applied in water analysis (**B**) of a laboratory-made portable capillary electrophoresis system with contactless conductivity detection [50]. In (**A**): (1) membrane pump, (2) valves, (3) splitter, (4) detector, (5) safety cage for application of high voltage, (6) pressurized air. In (**B**): recoding of the CE analysis of a tap water sample spiked with 1 μM NO_2_^−^. (a) Normal injection volume: 1 bar, 150 μL, splitting valve set to 0.15, 4 s. (b) Large volume injection: pressure, 1 bar; sample loop, 150 μL; splitting valve set to 0.10; injection time, 10 s. CE conditions: capillary—60 (36) cm, 50 μm I.D., BGE—12 mM His adjusted to pH 4 with AcOH, 2 mM of 18-crown-6, V: +15 kV. (Reproduced under permission from ACS).

Another type of portable CE-C^4^D instrument was designed with the use of automated injection [50]. This portable instrument can operate continuously for 9 h in battery-powered mode. The system can be optimized for different samples, and very fast separations of Cl^−^, NO_3_^−^, SO_4_^2−^ (within 17 s) and high reproducibility were reported. Its use in the field was demonstrated for the determination of phosphates at a sewage treatment plant and a tap water sample spiked with 1 μM NO_2_^−^ (Figure 10). For a 4s sample injection time, chloride, nitrate, and sulfate were satisfactorily separated, but a peak for nitrite was not detected due to its concentration being below the detection limit. The signal for nitrite can be also detected by increasing the injection time up to 10 s and increasing the pressure.

A fully automated, portable capillary electrophoresis analyzer can be also compatible with different detectors—ESI-MS, C^4^D and LIF—for potential in-situ deployment in spaceflight missions and exploration [108]. The implementation of these detectors can enable the analysis of a wide range of organic compounds potentially indicative of life, as well as inorganic compounds that can serve as indicators of habitability. Combining microfluidic and pneumatic circuits with rotary valves ensures full automation and good repeatability.

## 6. Conclusions and Perspectives

Capillary electrophoresis is an analytical technique which exhibits numerous advantages from the point of view of potential applications in the analysis of waters of different origin. The use of appropriate detectors allows its application in the determination of numerous organic compounds, and of inorganic ions, which is the subject of this review of original works published in recent decades. The possibility of the application of preconcentration in off-line mode, and especially on-line (in-capillary) provides opportunities of the application for the determination of trace level analytes. CE instrumentation can be more easily miniaturized to a microfluidic format than high-performance chromatographic HPLC or ion-chromatography instrumentation.

In the determination of inorganic ions the application of conductivity detection, particularly contactless C^4^D detection, which responds to all ions is especially suitable. The simultaneous determination of anions and cations can be carried out in the same capillary, and usually in shorter time than ion chromatography. Moreover, CE instrumentation is generally less expensive than ion chromatography, and the scaling down of the instruments is easier in the case of CE than for ion chromatography.

Admittedly, this technique is not commonly employed in routinely for water quality control, despite the development of numerous CE methods for water analysis and the aforementioned advantages. This seems to be mostly due to much wider availability of commercial instrumentation for HPLC than for CE, which can be easily adapted for ion chromatography without purchasing a dedicated ion chromatograph. The more difficult optimization of experimental conditions for CE determinations may be a second reason which inhibits the routine application of CE, compared to chromatography, but this is a subjective opinion. It can be assumed, however, that the publication of reviews on that subject may provide a valuable impact by means of widening the interest in the use of CE for water analysis. Several trends in CE development for water analysis remain to be emphasized. One of them is the design of multi-dimensional instrumental setups. For instance, the IC x CE-MS system was developed for simultaneous determinations of anions and cations, and has been employed for the speciation of arsenic [109]. There is also A review on systems combining chromatography with electrophoresis [110]. In a search for new detection techniques, a surface-enhanced Raman scattering-based microfluidic CE system has been reported, with applications including DNA-functionalized substrate use in the determination of Hg(II) in sewage water with an impressive LOD of 1 pM [111]. One example of efficient analyte preconcentration is electrokinetic supercharging, which obtained an enrichment factor of 500,000 in the determination of rare-earth metal ions [112]. This is certainly among the valuable ways to improve the limits of detection, e.g., in the determination of inorganic analytes in sea waters [113]. In the increasing applications of CE microfluidics (with some attempts to describe it as “nano-capillary electrophoresis” [114]), and the improvements of in-capillary sample processing methods, one can notice some perspectives of new routine applications of CE in water analysis.

## Figures and Tables

**Table 1:** Applications of capillary electrophoresis methods with various detections in water analysis.

Analyte(s)	Sample	Sample Pretreatment	Separation Conditions	Detection, LOD	First Author, Year of Publication	Ref.
Anions (Cl^−^, SO_4_^2−^)	Highly saline oilfield waters	Dilution	Coated capillary: 60 cm, 50 μm I.D. BGE: 50 mM TRIS, 30 mM SDS, 5% MeOH and 26 mM CrO_3_ (pH 6.7) V: −10 kV	Indirect UV 280 nm Cl^−^ 2,61 mg⋅L^−1^, SO_4_^2−^ 2,98 mg⋅L^−^^1^	Donkor, 2015	[22]
Anions (Br^−^,Cl^−^, NO_3_^−^, SO_4_^2−^)	Oilfield waters	Dilution	Coated capillary: 60.2 (50) * cm, 50 μm I.D. BGE: 10 mM HPTS, 0.4 M formic acid (pH 2) V: −22.5 kV	Indirect FL—LED 405/520 nm 0.4 mg·L^−1^ for SO_4_^2−^, 1.4 mg·L^−1^ for Cl^−^	Pei, 2015	[35]
Anions (ClO_4_^−^, Cl^−^, NO_3_^−^, SO_4_^2−^)	Tap and well water	μ-EME with FLM	Capillary: 46 (13) cm, 25 μm I.D. BGE: 10% AcOH (pH 2.2) V: −20 kV	C^4^D EF 30	Kuban, 2014	[43]
Anions (ClO_4_^−^, Cl^−^, NO_3_^−^, SO_4_^2−^, NO_2_^−^)	Surface, rain tap, snow, potable waters	EME	Capillary: 60 (45) cm, 50 μm I.D. BGE: 7.5 mM His and 40 mM AcOH (pH 4.1) V: −30 kV	C^4^D 1 mg⋅L^−1^ tap water 0.25–0.35 mg·L^−1^ for environmental and potable waters	Kiplagat, 2011	[53]
Anions (BF_4_^−^, ClO_4_^−^, PF_6_^−^, I^−^, NO_3_^−^, Br^−^, Cl^−^)	Tap water		Capillary: 60 (50) cm, 75 μm I.D. BGE: DMF–AcOH V: −14 kV	C^4^D 0.83–3.83 μM	Tian, 2014	[62]
Anions (total Re, Re(IV), and Re (VII))	Groundwater	Reduction of Re(VII) to Re(IV)	Capillary: 104 cm, 75 μm I.D. BGE: 10 mM K_2_CO_3_ (pH 11) V: 27 kV	ICP-MS 0.02 μg·L^−1^ for TRe, 0.01 μg·L^−1^ for Re(VII)	Zhou, 2021	[69]
Anions, chlorine containing (ClO_3_^−^,ClO_2_^−^, ClO_4_^−^, Cl^−^)	Drinking and swimming pool waters		Capillary: 90 cm, 50 μm I.D. BGE: 100 mM ammonium formate (pH 6.5) V: −20 kV	MS 0.25–2.1 mg⋅L^−1^ for ClO_3_^−^ 6 μg·L^−1^ (for large sample volume)	Gaspar, 2019	[72]
Anions, sulfur containing (SO_4_^2−^, S_2_O_3_^2−^, S_3_O_6_^2−^,vS_4_O_6_^2−^, S_5_O_6_^2−^)	Tailings pond water	FASS	Capillary: 48.5 (40.0) cm, 50 μm I.D. BGE: 2.00 mM PMA, 0.80 mM HMOH (pH 8.0) V: −20 kV	Indirect UV 350 nm 0.02–0.12 mg·L^−1^	Pappoe, 2014	[21]
Anions (Cl^−^, NO_3_^−^, SO_4_^2−^) Cations (Ca^2+^, K^+^, Mg^2+^, Na^+^, NH_4_^+^)	Cold and hot tap waters, well water		Capillary 60 (51.5) cm, 50 µm I.D. BGE1: 9 mM pyridine, 12 mM glycolic acid, and 5 mM 18-crown-6 ether (pH 3.6) BGE2: PMA (pH 7.7) V: ±20 kV	Indirect UV (DAD) 220, 254 nm 0.05–0.10 mg·L^−1^ (cations), 0.10 mg·L^−1^ (anions)	Fellah, 2017	[17]
Anions (Cl^−^, NO_3_^−^, NO_2_^−^, SO_4_^2−^) Cations (K^+^, Na^+^, Ca^2+^, Mg^2+^, NH_4_^+^) Phosphate As(III)	Ground and surface waters		Capillary: 65 (49) cm, 25 μm I.D. BGE1: 12 mM His,2 mM 18-crown-6 (pH 3.7) V: 15 kV; Capillary: 52 (36) cm, 25 μm I.D. BGE2: 12 mM His (pH 4.0 AcOH), V: −15 kV; Capillary: 52 (36) cm, 25 μm I.D. BGE3: 1 mM His (pH 3.5 AcOH) V: −15 kV; Capillary: 60 (52) cm, 25 μm I.D. BGE4: 12 mM MES, 21 mM Arg, 30 mM CTAB (pH 8.9) V: −20 kV	C^4^D 2.5–10 μM	Duong, 2015	[47]
Anions (Cl^−^, NO_3_^−^, SO_4_^2−^, NO_2_^−^, F^−^, H_2_PO_4_^−^) Cations (NH_4_^+^, K^+^, Ca^2+^, Na^+^, Mg^2+^, Li^+^)	Tap water sewage	Portable	Capillary: 50 (36) cm, 50 μm I.D. BGE: 12 mM His, 2 mM 18-crown-6 (pH 4 AcOH) V: ±15 kV	C^4^D 1.5–17 μM	Mai, 2013	[50]
Anions (Cl^−^, NO_3_^−^, SO_4_^2−^) Cations (Na^+^, K^+^, NH_4_^+^, Ca^2+^, Mg^2+^, Mn^2+^, Fe^2+^)	Sediment porewater	Portable	Capillary: 55 cm, 50 μm I.D. BGE: 11 mM His, 50 mM AcOH, 1.5 mM 18-crown-6, 0.1 mM citric acid V:±15 kV	C^4^D Cations 0.46–1.55 μM Anions 0.28–0.98 μM	Torres, 2013, 2014	[8,52]
Anions (Cl^−^, NO_3_^−^, SO_4_^2−^, NO_2_^−^, F^−^, PO_4_^3−^) Cations (NH_4_^+^, Na^+^, K^+^, Ca^2+^, Mg^2+^)	Tap water Lake water	On-site ion analyzer	Coated capillary: 60 (50) cm, 25 μm I.D. BGE: 400 mM Bis-Tris, 400 mM MOPS and 2 mM 18-crown-6 (pH 6.8) V: ±15 kV	C^4^D 2.1 μM (K^+^) 6.8 μM (PO_4_^3−^)	Li, 2021	[58]
Arsenic speciation (As (III), As (V), DMA, MMA, AsB, AsC, 3-NHPAA, 4-NPAA, o-ASA and p-UPAA)	Groundwater		Capillary: 100 cm, 50 μm I.D. BGE: 12 mM NaH_2_PO_4_ and 8 mM HBO_3_ (pH 9.20) V: 30 kV	ICP-MS 19–65 fg As	Liu, 2013	[67]
Arsenic speciation (As(III), As(V), MMA, DMA)	Tap water	SDME	Capillary: 60 (50) cm, 25 μm I.D. BGE: 15 mM phosphate buffer (pH 10.6) V: 25 kV	UV 200 nm As(III)–0.2 μM, DMA–0.7 μM, MMA–0.1 μM, As(V)–0.2 μM	Cheng, 2013	[32]
Arsenic speciation (As(III), As(V), MMA, DMA)	Spring water	CF-EKS	Capillary: 60 (50) cm, 50 μm I.D., μSiL-FC coated BGE: phosphate buffer (pH 9.6) LE and CHES (pH 9.6) TL V: −20 kV	UV 200 nm 0.08–0.3 μg·L^−1^ As EFs 6300–45,000-fold	Lee, 2018	[33]
Arsenic speciation (DMA, MMA, As(III), As(V))	Grandwater, well water, bottled water	Partial evaporation of the sample (50:1)	Capillary: 72 cm, 50 μm I.D. BGE: 57 mM HFIP (pH 10.3) V: 30 kV	ESI-MS 0.02–0.04 μg·L^−1^ (As)	Dominguez-Alvarez, 2020	[70]
Arsenic speciation (As(III), As(V))	River sediment	Microwave extraction	Capillary: 60 cm, 100 μm I.D. BGE: 25 mM NaH_2_PO_4_—Na_2_HPO_4_ (pH 6.6) V: 25 kV	HG-ETAAS As(III)–135 ng·g^−1^ As(V)–160 ng·g^−1^	Deng, 2013	[74]
Arsenic speciation (As(III), As(V), MMA, DMA, AsB) Selenium speciation (Se(IV), Se(VI), SeCys, SeMet, MeSeCys)	Groundwater, tap water		Capillary: 60 cm, 75 μm I.D. BGE: 6 mM NaH_2_PO_4_, 9 mM H_3_BO_3_ (pH 9.0) V: 25 kV	ICP-MS 0.11−0.37 μg·L^−1^ for arsenic compounds 1.33−2.31 μg·L^−1^ for selenium species	Liu, 2014	[68]
Aresenic speciation (As(III) and As(V)]) Selenium speciation (Se(IV) and Se(VI)) Bromate	Drinking water	PAEKI	Capillary: 110 cm, 50 μm I.D. BGE: 20 mM ammonium carbonate (pH 9.2) V: 30 kV + 50 mbar	ESI-MS/MS 1–3 μg·L^−1^	Zhang, 2011	[71]
Bromate	Drinking water	On-line ITP	Capillary: 24 (18) cm, 300 μm I.D. BGE: 50 mM phosphate, 20 mM glycine (pH 2.0), 0.1% MHEC, constant current mode 50 μA	UV 200 nm 0.6 μg·L^−1^	Marak, 2012	[24]
Bromate	Tap water	FASS	OT-CEC (coated with TMAPL) Capillary: 50 (41.5) cm 50 μm I.D. BGE: 20 mM Tris–14 mM HClO_4_ (pH 7.80) V: −20kV	DAD 235 8 μg·L^−1^	Guo, 2013	[25]
Bromate	Tap and bottled water	EME	Capillary: 80 (73) cm, 25 μm I.D. BGE: 300 mM AcOH, V: −18 kV	C^4^D 0.12 μg·L^−1^	Zhang, 2016	[45]
Cations (NH_4_^+^, K^+^, Ca^2+^, Na^+^, Sr^2+^, Cd^2+^, Pb^2+^; Mg^2+^; Fe^2+^, Ni^2+^, Zn^2+^, Cu^2+^)	Mineral, tap and well waters	Dilution in the ratio 1: 10 for tap water	Coated capillary: 60 (50) cm, 75 μm I.D. BGE: 20 mM benzimidazole,75 mM AcOH, 0.6 mM 18-crown-6 (pH 4.22) V: 25 kV	Indirect UV 254 nm 0.015–0.10 mg·L^−1^	Kowalski, 2017	[18]
Cations (NH_4_^+^, K^+^, Ca^2+^, Na^+^, Mg^2+^, Pb^2+^)	Natural, and potable waters, wastewater		Capillary: 64.5 (56) cm, 75 μm I.D. BGE: 15 mM imidazole, 8 mM malonic acid, 2 mM 18-crown-6 ether, 10% *v*/*v* MeOH (pH 4.35) V: −20 kV	Indirect UV 214 nm 0.023–0.084 mg·L^−1^	Varden, 2017	[19]
Cations (K^+^, Na^+^, Ca^+2^, Mg^+2^)	Sea water	Dilution 1:10 (*v*/*v*)	Capillary: 50 (40) cm, 50 μm I.D. BGE: 200 mM 2,4,6-trimethylpyridine, 250 mM lactic acid, 5% *v*/*v* MeOH (pH 4.5) V:25 kV	Indirect UV 230 nm ~10 mg·L^−1^	Lancioni, 2021	[20]
Cations (Ca^2+^, Mg^2+^, Cu^2+^, Zn^2+^, Ni^2+^, Co^2+^, Mn^2+^, Cd^2+^, Pb^2+^)	River water	Derivatization	Capillary: 60 (46.5) cm, 50 μm I.D. BGE: 50 mM borate buffer (pH 10.09), 0.05% PB, 1.0 mM DOTA V: 20 kV	LIF low ng·L^−1^ levels	Saito, 2011	[34]
Cations (Mn^2+^, Cd^2+^, Zn^2+^, Co^2+^, Pb^2+^, Cu^2+^, Ni^2+^)	Drinking and sea water	EME	Capillary: 50 (37) cm, 50 μm I.D. BGE: 5.2 M AcOH V: 20 kV	C^4^D 1–2.6 nM	Silva, 2018	[41]
Cations (Na^+^, K^+^, Li^+^, Ca^2+^, Mg^2+^, NH_4_^+^)	Ocean and lake waters	Dilution	Capillary: 64(43) cm, 50 μm I.D. BGE: 5 M AcOH, 10 mM 18-crown-6 and 10% ACN V: 30 kV	C^4^D 1.0 μM	Ferreira, 2018	[42]
Cations (Mn^2+^, Cd^2+^, Zn^2+^, Co^2+^, Pb^2+^, Cu^2+^, Ni^2+^)	Tap water	EME	Capillary: 50 (37) cm, 50 μm I.D. BGE: 20 mM l-His and 17 mM AcOH (pH 5.6) V: 25 kV	C^4^D 25–200 nM	Kuban, 2011	[46]
Cations (Na^+^, K^+^, NH_4_^+^, Ca^2+^, Mg^2+^)	Porewater of lake sediment core	Micro-injection	Capillary: 60 (49) cm, 25 μm I.D., BGE: 30 mM MES/His (pH 6), 2 mM 18-crown-6 V: −30 kV	C^4^D 10 μM	Saiz, 2015	[51]
Cations (Na^+^, K^+^, Li^+^, Ca^2+^, Mg^2+^, NH_4_^+^)	Drinking water and soil extracts		Capillary: 126 × 8 mm I.D. channels and 49.1 cm in length BGE: 20 mM MES/His (pH 6.1), 2 mM 18-crown-6. V: 286 mV/cm	C^4^D NH_4_^+^–3.2 μM Na^+^–2 μM	Nakatani, 2019	[60]
Fluoride	Seawater	10-fold dilution/tITP	Capillary 87.4 (75) cm, 75 µm I.D. BGE: 5 mM PDC (pH 3.5), 0.03% *m*/*v* HPMC V: 23 kV	indirect UV 200 0.024 mg·L^−1^	Fukushi, 2018	[26]
Mercury speciation (Hg(II), MeHg, EtHg and PhHg)	Tap, sea and surface waters	DLLME	Capillary: 60.2 (50) cm, 75 μm I.D. BGE: of 75 mM boric acid, 10% (*v*/*v*) MeOH (pH 9.0) V: 20kV	UV 210 nm Hg(II)–1.5, MeHg–1.79, EtHg–1.62 and PhHg–0.23 μg·L^−1^ EFs 46, 102, 118 and 547	Yang, 2014	[27]
Mercury speciation Hg(II) MeHg, EtHg and PhHg	River and lake waters	HF-LLLME	Capillary: 48.5 (40) cm, 50 μm I.D. BGE: 35 mM borate buffer (pH 9.10) V: 25 kV	UV 200 nm Sub μg·L^−1^ level EF 2195	Li, 2015	[28]
Mercury speciation(Hg(II), MeHg, EtHg and PhHg)	River and lake waters	PT-LLLME and LVSS	Capillary: 64.5 (56) cm, 50 μm I.D. BGE: 35 mM borate buffer (pH 9.10) V: 25 kV	UV 200 nm Sub μg·L^−1^ level EFs up to 12,138	Li, 2011	[29]
Mercury speciation (Hg(II), MeHg, EtHg and PhHg)	River water	HF-LLLMME	Capillary: 48 cm, 75 μm I.D. BGE: 100 mM borate buffer (pH 9) V: 15 kV	UV 210 nm 0.07–1.0 μg·L^−1^ (as Hg) EFs: Hg(II)–103, MeHg–265, EtHg–511 and PhHg–683	Chen, 2013	[30]
Mercury speciation (Hg(II), MeHg, EtHg)	Tap water	DSPE and FASI	Capillary: 90 cm, 75 μm I.D. BGE: 50 mM H_3_BO_3_—12.5 mM Na_2_B_4_O_7_ (pH 9.20) V: 12 kV	ICP-MS 9–11 ng·L^−1^	Chen, 2016	[65]
Mercury speciation (Hg(II) and MeHg)	River water	-	Capillary: 16 cm, 75 μm I.D. BGE: 30 mM boric acid and 5% (*v*/*v*) MeOH (pH 8.6) V: 21 kV	ICP-MS 9.7 mg·L^−^^1^ MeHg 12.0 mg·L^−1^ Hg(II)	Li, 2011	[66]
Selenium speciation (Se(IV) and Se(VI))	River, spring, and tap waters	SPME and FASS	Capillary: 64.5 (50) cm, 75 μm I.D. BGE: 0.2 M Tris-phosphate (pH 2.5), 0.1 mM CTAB V: −20 kV	UV 200 nm Se(IV)–57 ng·L^−^^1^ EF 41367 Se(VI)–ng·L^−1^ 71 EF 61935	Duan, 2012	[31]
Selenium speciation (Se(IV), Se(VI)m SeMet, SeCys_2_)	Wastewater	MSPE	Capillary: 80 cm, 75 μm I.D. BGE: 20 mM phosphate buffer (pH 10.6), 0.2 mM CTAB V: 25 kV	ETAAS Se(VI)–0.18 μg·L^−1^, Se(IV)–0.17 μg·L^−1^	Yan, 2015	[73]

* value in parentheses—the effective length of capillary; C^4^D—capacitively-coupled contactless conductivity detection, CHES—N-Cyclohexyl-2-aminoethanesulfonic acid, CF-EKS—counter flow with electrokinetic supercharging, CTAB—cetyltrimethylammonium bromide, DMF—dimethylformamide, DLLME—dispersive liquid–liquid microextraction, DOTA—1,4,7,10-tetraazacyclododecanetetraacetate, DSPE—dispersive solid-–phase extraction, EME—electromembrane extraction, EF—enrichment factor, FASI—field-amplified sample stacking injection, FASS—field-amplified sample stacking, FL—fluorescence detection, LED—light emitting diode, FLM—free liquid membrane, His—histidine, HF—hollow fiber, HFIP—hexafluoro-2-propanol, HMOH—hexamethonium hydroxide, HPTS—8-Hydroxypyrene-1,3,6-trisulfonic acid, MHEC—methylhydroxyethylcelullose, HPMC—hydroxypropyl methylcellulose, tITP—transient isotachophoresis, LLLME—liquid–liquid–liquid microextraction, LLLMME liquid–liquid–liquid membrane microextraction, LVSS—large volume sample stacking, MES—2-(*N*-morpholino) ethanesulfonic acid, MOPS—3-(*N*-morpholino)propanesulfonic acid, MSPE—magnetic solid–phase extraction, OT-CEC—open tubular capillary electrochromatography, PAEKI—pressure-assisted electrokinetic injection, PB—polybrene, PDC—2,6-pyridinedicarboxylic acid, PMA—pyromellitic acid, PT—phase transfer, SDME—single drop microextraction, μSiL-FC—fluorocarbon polymer-coated fused silica capillary, SPME—solid phase microextraction, TMAPL—trimethylamine amination polychloromethyl styrene nanolatex.

**Table 2:** Capillary electrophoretic methods developed for simultaneous determination of inorganic anions and cations in waters.

Analyte(s)	Sample	System Configuration	Separation Conditions	Detection, LOD	First Author, Year of Publication	Ref.
Anion HPO_4_^2−^ Cation Ca^2+^	River water	On-column complexation	Capillary: 55.0 (48.5) * cm, 50 μm I.D. BGE: 10 mM PDCA, 0.75 mM TTAB (pH 7.0) V: −20 kV	UV 214 nm 5 μM for [Ca(PDCA)_2_]^2^^−^ 2 μM for HPO_4_^2^^−^	Wang, 2011	[23]
Anions (NO_3_^−^, SO_4_^2−^) Cations (K^+^, NH_4_^+^)	Fertiliser solution	Dual capillary system	Capillary: 10.5 (8.0) cm, 25 μm I.D. BGE: 500 mM AcOH, 20 mM Tris, 2 mM 18-crown-6 (pH 3.3) V: ±10 kV	C^4^D 6.9 μM K^+^ 10.6 μM NH_4_^+^	Opekar, 2016	[44]
Anions (Cl^−^, NO_3_^−^, SO_4_^2−^, NO_2_^−^, F^−^, PO_4_^3−^) Cations (Na^+^, K^+^, Li^+^, Ca^2+^, Mg^2+^, NH_4_^+^)	Creek water	SIA-CE	Capillary: 60 (35) cm, 50 μm I.D. BGE:12 mM His, 2 mM 18-crown-6 (pH 4) V: ±20 kV	C^4^D Anions: 0.7–2.0 μM Cations: 13–40 μM	Mai, 2010	[48]
Anions (NO_3_^−^, NO_2_^−^, Cl^−^, Br^−^, F^-^, SO_4_^2−^, PO_4_^3−^, ClO_4_^−^, ClO_3_^−^, CrO_4_^2−^, MoO_4_^2−^) Cations (NH_4_^+^, K^+^, Na^+^, Mg^2+^, Ca^2+^, Mn^2+^, Zn^2+^, Sr^2+^, Cd^2+^, Fe^2+^)	Tap and process waters	Dual capillary—SI	LPA coated capillaries: Cations—55 (35) cm, 50 μm I.D., Anions—50 (28) cm, 50 μm I.D., BGE: 50 mM AcOH,10 mM His, 2.5 mM 18-crown-6 (pH 4.2) V: ±30kV	C^4^D Anions: 5–61 μg·L^−^^1^ Cations: 13–40 μg·L^−^^1^	Gaudry, 2013	[54]
Anions (NO_3_^−^, NO_2_^−^) Cation (NH_4_^+^)	Contaminated groundwater	Dual capillary system	Capillary: 55 (40) cm, 50 μm I.D. BGE: 12 mM His, 2 mM 18-crown-6 (pH 4) V: ±15 kV	C^4^D 5.0 μM NH_4_^+^ 6.0 μM NO_3_^−^ 7.5 μM NO_2_^−^	Pham, 2014	[55]
Anions (Cl^−^, NO_3_^−^, SO_4_^2−^) Cations (K^+^, Na^+^, Mg^2+^, Ca^2+^)	Mineral and tap waters	GEMBE	Capillary: 5.0 cm, 15 μm I.D. BGE: 100 mM AcOH and 10 mM His (pH 3.76) V: 20 kV	C^4^D Anions: 0.34–1.13 mg·L^−^^1^ Cations: 0.76–3.09 mg·L^−^^1^	Flanigan, 2010	[57]
Anions (Cl^−^, NO_3_^−^, SO_4_^2−^) Cations (NH_4_^+^, K^+^, Na^+^, Ca^2+^, Mg^2+^, Mn^2+^, Zn^2+^, Cd^2+^, Ba^2+^	Tap water	SIA/Dual single-end injections	Capillary: 50 cm, 10 μm I.D, *L*_eff_ for cations—43 cm and for anions—35 cm; BGE: 12 mM His, 2 mM 18-crown-6 (pH 4) V: 20 kV	Dual C^4^D Anions: 1.5–2.0 μM Cations: 0.3–1.5 μM	Mai, 2012	[58]
Anions (NO_3_^−^, NO_2_^−^) Cation (NH_4_^+^)	Water quality monitoring after wastewater treatment	SIA-CE	Capillary: 68.0 cm, 20 μm I.D. BGE: 100 mM His, 100 mM MES, 0.13 mM CTAB, 1.5 mM 18-crown-6 (pH 6) V: 24 kV	C^4^D 0.03 mg⋅L^−^^1^ NO_2_^−^, 0.08 mg⋅L^−^^1^NO_3_^−^, 0.11 mg⋅L^−^^1^ NH_4_^+^	Fuiko, 2019	[61]
Anions (Cl^−^, Br^−^, NO_3_^−^, NO_2_^−^, SO_4_^2−^, PO_4_^3−^) Cations (NH_4_^+^, K^+^, Na^+^, Li^+^, Mg^2+^, Ca^2+^)	Drinking water (domestic well)	DOI dual opposite end injection	PVA coated capillary: 60 cm, 50 μm I.D. BGE: 15 mM PMA, 10 mM citric acid, 2 mM 18-crown-6 (pH 3.70 adjusted with His) V: 30 kV	C^4^D Anions: 0.076–2.51 mg·L^−^^1^ Cations: 0.075–2.33 mg·L^−^^1^	Neaga, 2014	[62]
Anions (Cl^−^, NO_3_^−^, SO_4_^2−^) Cations (NH_4_^+^, Na^+^, K^+^, Ca^2+^, Mg^2+^, Mn^2+^, Zn^2+^, Cu^2+^)	Sediment porewater, well and mining pond water	Dual-channel portable	Capillary: 90 (80) cm, 25 μm I.D. BGE for anions: 7.5 mM His and 40 mM AcOH BGE for cations: 9 mM His, 4.6 mM lactic acid, 25 mM AcOH, 1mM 18-crown-6 V: ±25 kV	C^4^D Anions: 10–12 μM Cations: 2.8–4.8 μM	Koenka, 2016	[63]
Anions (Cl^−^, NO_3_^−^, SO_4_^2−^) Cations (Na^+^, K^+^, NH_4_^+^, Ca^2+^, Mg^2+^)	Mineral and tap waters	CFM	Capillary: 35.0 (20 and 15) cm, 25 μm I.D. BGE: 18 mM His, 130 mM malic acid, 100 mM DDAPS, 3 mM18-crown-6 (pH 3.6) V: 30 kV	C^4^D Anions: 0.4–0.6 mg·L^−^^1^ Cations: 0.4–0.6 mg·L^−^^1^	Yamamoto, 2019	[85]

* value in parentheses—the effective length of capillary; C^4^D—capacitively coupled contactless conductivity detection, CE—capillary electrophoresis, CFM—capillary filling method, CTAB—cetyltrimethylammonium bromide, DDAPS—dodecyl-*N*,*N*-dimethyl-3ammonio-1-propanesulfonate, DOI—dual opposite end injection, GEMBE—gradient elution moving boundary electrophoresis, *L*_eff_—effective length, PVA—polyvinyl acetate, LPA—linear polyacrylamide, SIA—sequential injection analysis, MES—2-(*N*-morpholino) ethanesulfonic acid, TTAB—tetradecyltrimethylammonium bromide, PDCA—2,6-pyridinedicarboxylic acid.6. Application of Microfluidic CE Systems.

**Table 3:** Application of microfluidic chips for capillary electrophoretic analysis of waters.

Analyte(s)	Sample	Chip	Separation Conditions	Detection, LOD	First Author, Year of Publication	Ref.
Ammonium	Wastewater	CZE and ITP-CZE PMMA	Channel C1—59 mm × 0.2 mm–0.5 mm × 0.14 mm–0.2 mm; channel C2—56 mm × 0.2 mm–0.5 mm × 0.14 mm–0.2 mm) BGE1: LE: 1.25 mM ethylenediamine, 3.75 mM acetic acid, 50 mM 18-crown-6, 0.1% *v*/*v* PEG, pH 5.4; TE: 10 mM sodium acetate, 10 mM AcOH, 0.1% *v*/*v* PEG (pH 4.8), I = 15 µA BGE2: 50 mM AcOH, 25 mM 18-crown-6 and 4 mM tartaric acid, 0.1% *v*/*v* PEG (pH 3.0) I = 25 µA	C^4^D 20 µg·L^−1^ CZE 40 µg·L^−1^ ITP-CZE	Luc, 2011	[99]
Anions (Cl^−^, NO_3_^−^, SO_4_^2−^)	Drinking water	PMMA	Channel: 85 (65) mm, 50 × 50 µm BGE: 18 mM aspartate (pH 4.15), 0.1% MHEC, 5.94 mM Bis-tris propane, 100 mM DDAPS I: 40 μA	C^4^D 40–120 µg·L^−1^	Masar, 2012	[97]
Anions (Cl^−^, NO_3_^−^, F^−^, SO_4_^2−^, SCN^−^, PO_4_^3−^)	Mineral water, tap water	PDMS	Channel: 65 mm, 100 µm BGE: 50 mM MES/His (pH 6.0), 0.5% PVP V: 2 kV	C^4^D with in-plane electrodes 3.6–14.7 μM	Koczka, 2016	[100]
Anions (Br^−^, Cl^−^, NO_3_^−^, NO_2_^−^, F^−^, SO_4_^2−^) Cations (NH_4_^+^, K^+^, Na^+^, Li^+^, Ca^2+^, Mg^2+^)	Bottled drinking water	PMMA	Channel: 85 (65) mm, 50 × 50 µm BGE: 30 mM MES/His (pH 6), 2mM 18-crown-6 V: ±4 kV	C^4^D dual top–bottom cell 0.3 µM cations, 0.15 µM anions	Mahabadi, 2010	[101]
Anions (Cl^−^, NO_3_^−^, NO_2_^−^, SO_4_^2−^)	Aquarium, river water	Borosilicate glass	Channel: 33 mm, 10 × 100 µm BGE: 30 mM latic acid and 15 mM His (pH 3.8) V: −1.0 kV	C^4^D 2.0 to 4.9 μM	Freitas, 2016	[102]
Cations (K^+^, Na^+^, Li^+^, Ca^2+^, Mg^2+^, Zn^2+^, Cd^2+^, Cu^2+^)	River water	PDMS/PET	Channel: 50 mm, 50 × 50 µm BGE1: 10 mM MES, 10 mM His BGE2: 0.1 M acetic buffer pH 4.0 V: 5kV	C^4^D with ITO-coated films electrodes 5.8 μM K^+^, 8.0 μM Na^+^, 14.3 μM Li^+^	Yan, 2015	[98]
Cations (Ag^+^, Hg^2+^)	Tap water and river water	Quartz	Channel: 23 mm, 104 × 48 μm BGE: phosphate buffer saline (pH 7.4) V: 250 V	LED LIF 0.038 nM Ag^+^, 0.054 nM Hg^2+^	Chen, 2019	[105]
Nitrite	Well water	PDMS	Channel: 50 mm, 15 × 15 μm BGE: 5 mM phosphate (pH 7.5), 200 mM CTAB V: −1.0 kV	Amperometric detection pencil graphite electrode 2.8 μM	Da Silva, 2017	[103]
Nitrite	Drinking water	PDMS	Channel: 50 mm, 15 × 15 μm BGE: 5 mM phosphate (pH 6.85), 200 mM CTAB V: −1.2 kV	Amperometric detection screen-printed carbon-based electrode 8.2 μM	Petroni, 2017	[104]
Perchlorate	Drinking water	PDMS	Channel: 20 mm, 50 × 50 µm BGE:10 mM nicotinic acid, 1.0 mM TDAPS (pH 3.6); V: −700 V	C^4^D 5.6 ± 1.7 µg·L^−1^	Gertsch, 2010	[96]

C^4^D—capacitively coupled contactless conductivity detection, CTAB—cetyltrimethylammonium bromide, DDAPS—dodecyl-*N*,*N*-dimethyl-3ammonio-1-propanesulfonate, His—histidine, ITO—indium tin oxide; MES—2-(*N*-morpholino) ethanesulfonic acid, MHEC—methylhydroxyethylcellulose, PDMS—poly(dimethylsiloxane), PET—poly-(ethylene terephthalate), PMMA polymethylmethacrylate, TDAPS—*N*-tetradecyl-*N*,*N*-dimethyl-3-ammonio-1-propane sulfonate.7. Portable CE Systems for Water Analysis.
